# Characterizing epithelial‐mesenchymal transition‐linked heterogeneity in breast cancer circulating tumor cells at a single‐cell level

**DOI:** 10.1002/1878-0261.70132

**Published:** 2025-10-05

**Authors:** Justyna Topa, Julia Richert, Tomasz Stokowy, Alicja Staśczak, Mariusz Szajewski, Maciej Ciesielski, Petra M. Grešner, Bartłomiej Tomasik, Łukasz Arcimowicz, Agnieszka Stankiewicz, Grażyna Suchodolska, Elżbieta Senkus, Wiesław Kruszewski, Anna J. Żaczek, Aleksandra Markiewicz

**Affiliations:** ^1^ Laboratory of Translational Oncology Intercollegiate Faculty of Biotechnology University of Gdańsk and Medical Univeristy of Gdańsk, Medical University of Gdańsk Poland; ^2^ International Centre for Cancer Vaccine Science University of Gdańsk Poland; ^3^ Scientific Computing Group, IT Division University of Bergen Norway; ^4^ Interdisciplinary Pomeranian Center for Digital Medicine Medical University of Gdańsk Poland; ^5^ Department of Systems Biology and Engineering, Faculty of Automatic Control, Electronics and Computer Science Silesian University of Technology Gliwice Poland; ^6^ Biotechnology Centre Silesian University of Technology Gliwice Poland; ^7^ Department of Oncological Surgery Gdynia Oncology Centre, Pomeranian Hospitals Gdynia Poland; ^8^ Department of Oncological Surgery, Faculty of Health Sciences with the Institute of Maritime and Tropical Medicine Medical University of Gdańsk Gdynia Poland; ^9^ Centre of Biostatistics and Bioinformatics Medical University of Gdańsk Poland; ^10^ Centre for Experimental Cardiooncology Medical University of Gdańsk Poland; ^11^ Department of Oncology and Radiotherapy, Faculty of Medicine Medical University of Gdańsk Poland; ^12^ Research Centre for Applied Molecular Oncology Masaryk Memorial Cancer Institute Brno Czech Republic

**Keywords:** breast cancer, circulating tumor cells, epithelial–mesenchymal transition, metastasis, RNA‐Seq, single cell transcriptomics

## Abstract

Epithelial–mesenchymal transition (EMT) generates heterogeneity in circulating tumor cells (CTCs), affecting their biological properties and hampering their detection. This limits our understanding of the mechanisms underlying hematogenous dissemination, especially in early breast cancer (BC), where CTCs are rare. Here, we aimed to detect CTCs with different EMT statuses from BC patients. CTCs in blood samples from 107 BC patients were evaluated using immunomagnetic depletion and multi‐marker immunofluorescence (EpCAM, E‐cadherin, MCAM, cell surface vimentin, CD31, CD45), followed by single‐cell transcriptomics. CTCs were detected in 51.9% of therapy‐naïve early BC cases, with 3.8% showing only epithelial CTCs (eCTCs), 5.8% epithelial‐mesenchymal (emCTCs), 26.0% mesenchymal (mCTCs), and 16.3% mixed phenotypes. CTC heterogeneity was more frequent in triple‐negative (86%) than in luminal BC (17%, *P* = 0.008). Lymph node involvement strongly predicted dissemination of all CTC phenotypes, while tumor size correlated with mCTC abundance. Single‐cell RNA sequencing revealed downregulation of ribosomal genes and translation inhibition in CTCs with mesenchymal features, linked to mTORC1 signaling. Findings were also validated in an independent dataset, highlighting vulnerabilities in CTCs during dissemination.

## Graphical abstract

In over 50% of non‐metastatic breast cancer patients, circulating tumor cells (CTCs) along the whole epithelial‐mesenchymal transition spectrum are detected. Total CTC number and individual phenotypes relate to aggressive disease characteristics, including lymph node involvement and higher tumor proliferation. At the single‐cell level, mesenchymal CTCs (in comparison to epithelial CTCs) have decreased translation, which might result from decreased mTORC1 signaling.

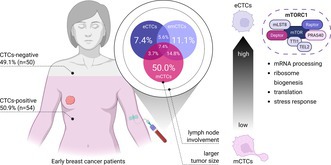

**Abbreviations**
95% CI: 95% confidence intervals
ACTB: actin beta
AIC: Akaike Information Criterion
APR: adaptive pausing response
ATCC: American Type Culture Collection
ATF4: activating transcription factor 4
avg.AIC: average AIC
avg.logLik: average logLik
avg.RMSE: average cross‐validated RMSE
BC: breast cancer
BSA: bovine serum albumin
CB: coating buffer
CDH1: cadherin 1
CK19: cytokeratin 19
Cq: cycle of quantification
csVIM: cell surface vimentin
CTCs: circulating tumor cells
CYFIP1: cytoplasmic FMR1 interacting protein
DAPI: 4′,6‐diamidino‐2‐phenylindole dihydrochloride
DEGs: differentially expressed genes
DMEM: Dulbecco's modified Eagle's medium
ECAD: E‐cadherin
eCTCs: epithelial CTCs
EEF1A1: eukaryotic translation elongation factor 1 alpha 1
emCTCs: epithelial‐mesenchymal CTCs
EMT: epithelial‐mesenchymal transition
EpCAM: epithelial cell adhesion molecule
ER: estrogen receptor
FBS: fetal bovine serum
FC: fold change
FDR: false discovery rate
GAPDH: glyceraldehyde 3‐phosphate dehydrogenase
GSEA: gene set enrichment analysis
HER2: human epidermal growth factor receptor type 2
IQR: interquartile range
IRRs: incidence rate ratios
ISR: integrated stress response
ISRIB: integrated stress response inhibitor
KRT19: keratin 19
logLik: log‐likelihood
MCAM: melanoma cell adhesion molecule
mCTCs: mesenchymal CTCs
MET: mesenchymal‐epithelial transition
MLST8: mTOR‐associated protein, LST8 homolog
MTOR: mammalian target of rapamycin
NCs: normal cells
NES: normalized enrichment score
PAI1: plasminogen activator inhibitor 1
PBMCs: peripheral blood mononuclear cells
PBS: phosphate‐buffered saline
PECAM1: platelet and endothelial cell adhesion molecule 1
PLS3: plastin 3
PLTs: platelets
pNCs: potential NCs
PR: progesterone receptor
PTPRC: protein tyrosine phosphatase receptor type C
RBCs: red blood cells
RMSE: root mean squared error
RPs: ribosomal proteins
RPTOR: regulatory‐associated protein of MTOR complex
RQ: relative quantity
RR: recovery rate
SD: standard deviation
SERPINE1: serine proteinase inhibitor family E member 1
tCTCs: true CTCs
TNBC: triple‐negative BC
tNCs: true NCs
VIM: vimentin

## 1 Introduction

Breast cancer (BC) remains the most prevalent cancer type in women and the leading cause of cancer‐related deaths in women worldwide [1]. The direct cause of cancer‐related death is distant metastases, which are seeded by circulating tumor cells (CTCs). These cells detach from the primary tumor and disseminate throughout the body via blood and lymphatic vessels. CTC counts are associated with prognostic value in early and metastatic BC and a potential marker facilitating the choice of treatment strategy [2]. This underlines the importance of CTC detection and analysis in BC management.

CTCs exhibit significant diversity in their phenotypes, and one of the factors introducing such heterogeneity is epithelial–mesenchymal transition (EMT), a physiological process hijacked by cancer cells [3]. As a result of EMT, cells may achieve enhanced migratory potential, increase resistance to apoptosis, improve survival in a hostile environment, and acquire stem cell‐like properties [3, 4, 5, 6]. These characteristics collectively support disease progression and metastasis formation. Evidence of EMT activation in disseminating cancer cells is common—mesenchymal CTCs (mCTCs) are detected in 35.5% of non‐metastatic and 90% of metastatic BC patients before treatment [7]. While epithelial CTCs (eCTCs; as detected by CellSearch®) are strongly associated with poor prognosis, changes in the EMT status of the CTCs provide additional valuable insight into disease progression. The mCTCs fraction often increases during disease progression, which is associated with unfavorable clinicopathological characteristics and shorter overall survival in both early and metastatic BC [7, 8, 9]. Therefore, better characterization of mCTCs is crucial for understanding their biology, identifying features that contribute to aggressive behavior, and discovering potential vulnerabilities that could be targeted to decrease tumor progression. However, identification of mCTCs is hampered due to EMT‐related changes in marker proteins that are used for CTCs isolation. Reduced expression of epithelial markers – E‐cadherin (ECAD) or epithelial cell adhesion molecule (EpCAM), together with a lack of consensus on specific mesenchymal markers for mCTCs identification, are particularly problematic when attempting to isolate intact, living CTCs, suitable for transcriptomic profiling. In such cases, intracellular markers, like well‐known EMT markers—vimentin (VIM) or transcription factors TWIST1, SNAIL (encoded by *SNAI1* gene), SLUG (encoded by *SNAI2* gene), and ZEB1 cannot be used for viable cell isolation. This is because cell membrane permeabilization is required, which results in RNA leakage. Instead, one must rely on surface markers for CTCs isolation and identification. Alternatively, RNA profiling methods applicable to methanol‐ or paraformaldehyde‐fixed cells have been described [10, 11] and have demonstrated good performance, including in the analysis of rare cell populations. In our study, we used the recently described cell surface presented form of VIM (csVIM), which enables the detection of mCTCs [8], and requires non‐fixed cells for optimal CTCs identification. csVIM is a form of intracellular VIM that is exposed on the surface of cancer cells with mesenchymal features [12], proving valuable for CTCs detection across a wide range of malignancies [12, 13, 14, 15, 16, 17, 18, 19, 20], including BC [8]. Notably, CTCs detection with csVIM has shown superior performance compared to the CellSearch® system in identifying BC patients who are not responding to treatment or experiencing disease progression [8]. Furthermore, the combined approach of detecting both mCTCs (using csVIM) and eCTCs (using CellSearch®) in the same sample has yielded significantly improved detection rates over either method used independently [8]. The melanoma cell adhesion molecule (MCAM) has also been identified as an effective marker for recovering spiked BC cell lines lacking EpCAM expression and CTCs in BC patients [21]. This marker has shown promise in enhancing the detection of CTCs in both early and metastatic BC patients [21, 22]. However, because MCAM can also be expressed on circulating endothelial cells, additional negative selection or exclusion markers, such as CD31 or CD34, are necessary to eliminate false positives and ensure accurate detection.

This phenotypic diversity necessitates the use of single‐cell analysis techniques to understand the varied aggressiveness of CTCs with different EMT phenotypes. CTCs heterogeneity is a key factor underlying the molecular mechanisms of tumor evolution [23, 24], metastasis [25], and treatment response or resistance [26]. Addressing these issues, we aimed to detect CTCs with different EMT status from BC patients using the developed protocol enabling their analysis on a single‐cell level [27]. The number and phenotype of CTCs was correlated with clinicopathological characteristics. Harvested single CTCs and normal cells (serving as controls) were subjected to Smart‐Seq2‐based reverse transcription and cDNA preamplification, and transcriptomic profiling (RNA‐seq) to uncover biological differences between them.

## 2 Materials and methods

### 2.1 Cell culture

Human BC cell lines MCF‐7 (RRID:CVCL_0031), SK‐BR‐3 (CVCL_0033), and MDA‐MB‐231 (CVCL_0062) were obtained from American Type Culture Collection (ATCC, cat. No. HTB‐22, HTB‐30, and HTB‐26, respectively). Cells were cultured in high‐glucose Dulbecco's modified Eagle's medium (DMEM; VWR, cat. No. VWRC392‐0414, Radnor, PA, USA) supplemented with 10% of fetal bovine serum (FBS; Gibco, cat. No. 10270–106, Waltham, MA, USA) and 1% of penicillin/streptomycin solution (Gene DireX, cat. No. CC502‐0100, Taoyuan, Taiwan) at 37 °C and 5% CO_2_. Cell cultures were routinely monitored for mycoplasma contamination to ensure experimental integrity; cell lines older than three years from the day of their purchase were authenticated using STR profiling at the Malopolska Center of Biotechnology.

### 2.2 Samples collection from BC patients and healthy volunteers

Peripheral blood samples were collected from 107 BC patients (including 104 early BC patients, 1 metastatic patient, and 2 non‐metastatic patients undergoing endocrine therapy) admitted to the University Clinical Centre in Gdansk between May 2019 and October 2020 and to the Gdynia Oncology Centre, Pomeranian Hospitals between February 2020 and May 2021. Statistical analysis between overall survival time, clinicopathological data, and CTCs number and phenotype was performed only for the 104 early BC patients, whereas RNA expression profiles were obtained from CTCs of early, metastatic, and treated BC patients, which is indicated in the corresponding figures and tables. The median survival time was 4.6 years (range 1.8–5.9 years).

The study patients had a median age of 61 years (mean ± SD 59.7 ± 13.1, range 25–86). Detailed clinicopathological characteristics of the patients are presented in Table S1. Tumor grade was assessed according to the modified Bloom and Richardson system. Given that this is the first study using csVIM to identify mesenchymal CTCs in non‐metastatic BC, patients across all molecular subtypes were included to evaluate the prevalence and heterogeneity of these CTCs across the BC spectrum, serving as a basis for future larger‐scale studies. ER and PR were deemed positive if the percentage of stained cells was ≥1% [28]. HER2 positivity was determined based on either a 3+ score in immunohistochemistry or a positive result of fluorescent or silver stain *in situ* hybridization. The molecular subtypes were defined according to the recommendations of the International Expert Consensus from St. Gallen (2013): luminal A – ER ≥1%, PR ≥20%, HER2‐negative, Ki67 < 20%; luminal B HER2‐negative – ER ≥1%, HER2‐negative, PR <20% and/or Ki67 ≥ 20%; luminal B HER2‐positive – ER ≥1%, any PR, HER2‐positive, any Ki67; HER2‐enriched (HER2+) – ER <1%, PR <1%, HER2‐positive, any Ki67; triple‐negative (TNBC) – ER <1%, PR <1%, HER2‐negative, any Ki67 [29, 30].

For evaluation of CTCs isolation, control blood samples were obtained from six healthy female volunteers with no prior cancer history. The median age of these control subjects was 29 years (mean ± SD 33.2 ± 7.8, range 28–48). All patients and healthy volunteers agreed to participate in the study and provided written informed consent. The study was performed according to the Declaration of Helsinki and received approval from the Bioethics Committee of the Medical University of Gdańsk (NKBBN/433/2017 and NKBBN/748/2019–2020).

### 2.3 CTCs' enrichment and isolation

For CTCs isolation, 5 mL of peripheral blood was collected into K_2_EDTA‐coated tubes (BD Vacutainer®, cat. No. BDAM367864, Franklin Lakes, NJ, USA) via venipuncture. The CTCs isolation method employed a multistep approach (Fig. S1). The first 1 mL of blood was discarded to eliminate potential contamination from epithelial cells and fibroblasts introduced during skin puncture. To remove platelets, the blood was centrifuged at 200 *g* at 21 °C for 10 min. The top layer of plasma was carefully removed, leaving about 1 mL of plasma above the buffy coat to avoid accidental aspiration of cellular fractions. The remaining sample was then diluted with 1× PBS at room temperature to a total volume of 9 mL, gently mixed, and carefully layered onto 4 mL of sterile Histopaque®‐1077 (Sigma Aldrich, cat. No. 10771, St. Louis, MO, USA) in a new 15 mL tube pre‐coated with coating buffer (CB; 2 mm ethylenediaminetetraacetic acid (EDTA), 1% FBS in 1× PBS). The sample was centrifuged at 400 *g* at 21 °C for 30 min without brake and acceleration. From this point onward, all subsequent steps were carried out on ice unless otherwise specified. The peripheral blood mononuclear cells (PBMCs) fraction was collected into a new CB‐pre‐coated 15 mL tube and brought to a total volume of 10 mL with cold (4 °C) 1× PBS. The sample was centrifuged at 450 *g* at 4 °C for 10 min to obtain a cell pellet. The PBMCs pellet was then suspended in 250 μL of blocking buffer (50 mm glycine, 5% bovine serum albumin (BSA, filtered)) and transferred into a CB‐pre‐coated 1.5 mL low‐bind tube (Ultra High Recovery Tubes, Starlab, cat. No. E1415‐2600, Milton Keynes, UK or Protein LoBind Tubes, Eppendorf, cat. No. 0030108116, Hamburg, Germany). Samples were incubated at 4 °C for 15 min on the HulaMixer™ Sample Mixer (Invitrogen, cat. No. 159‐20D, Waltham, MA, USA) and subsequently centrifuged at 400 *g* at 4 °C for 5 min. First, cells were stained with an antibody cocktail, followed by CD45‐positive cells magnetic depletion, as this sequence of steps was technically easier to perform and resulted in smaller losses of cell numbers. The supernatant was carefully removed, and 200 μL of freshly prepared in staining buffer (10 mm glycine, 1% BSA in 1× PBS, filtered) antibody mix targeting EpCAM (1:400; clone VU1D9, Alexa Fluor® 488‐conjugated, Cell Signalling Technology, cat. No. 5198, Danvers, MA, USA), ECAD (1:100; clone 67A4, Alexa Fluor® 488‐conjugated, Santa Cruz Biotechnology, cat. No. sc‐21 791), MCAM (1:200; clone P1H12, Alexa Fluor® 594‐conjugated, Santa Cruz Biotechnology, cat. No. sc‐18 837), csVIM (1:100; clone 84‐1, Texas Red‐conjugated, Abnova, cat. No. H00007431‐MT08, Taipei City, Taiwan), CD31 (1:100; clone WM‐59, Super Bright 436‐conjugated, eBioscience, cat. No. 62–0319‐42, San Diego, CA, USA), and 4′,6‐diamidino‐2‐phenylindole dihydrochloride (DAPI; 1:10000; Sigma Aldrich, cat. No. MBD0015) was added into the cell pellet and gently mixed. The sample was incubated at 4 °C for 30 min on the sample mixer., after which it was gently suspended and transferred to a pre‐coated 15 mL tube containing 125 μL of Dynabeads™ CD45 (Thermofisher, cat. No. 11153D, Waltham, MA, USA) in CB, washed according to the manufacturer's protocol. To collect any residual cells from the tube, the 1.5 mL tube was rinsed with 675 μL of CB and transferred into the 15 mL tube containing the stained PBMC fraction and Dynabeads™ CD45 (total volume of 1 mL). The samples were incubated for 30 min at 4 °C on the sample mixer. During this step, CD45‐positive cells were captured by the anti‐CD45 antibody‐coated magnetic nanoparticles. After 5 min of incubation (at 4 °C), 5 μL of anti‐CD45 antibodies (clone REA747, VioBlue®‐conjugated, Miltenyi Biotec, cat. No. 130–110‐637, Bergisch Gladbach, Germany) were added directly to the tube to achieve a final dilution of 1:200. Following the incubation, the tube was removed from the mixer, and the sample was diluted to a volume of 13 mL with cold (4 °C) CB. The sample was mixed gently with a Pasteur pipette and placed in the DynaMag™‐15 Magnet (Thermofisher, cat. No. 12301D) for 10 min (protected from light). CD45‐positive cells attached to the magnetic beads remained on the side of the tube while kept on the magnet, whereas CD45‐negative cells remained in the solution. The CTC‐enriched cell suspension was carefully transferred into a CB‐pre‐coated 50 mL tube and filled to a total volume of 25 mL with cold (4 °C) CB. The sample was centrifuged at 4 °C for 5 min; the supernatant was aspirated, and the cell pellet containing CTCs was resuspended in 100 μL of CB. The CTC‐enriched sample was then loaded onto a chamber slide (Nunc® Lab‐Tek® Chamber Slide™ system, Merck, cat. No. C7182, Darmstadt, Germany) and screened under a fluorescent microscope (ZEISS AxioVision 200; Zeiss, Oberkochen, Germany). The number and phenotype of putative CTCs were recorded based on the expression of epithelial (EpCAM and ECAD) and mesenchymal (MCAM and csVIM) markers, and the absence of endothelial (CD31) and hematopoietic (CD45) markers, as well as DAPI staining (lack of DAPI signal indicating living cells).

The specificity of immunofluorescence staining was assessed by identifying cells with CTCs markers (positive for either epithelial and mesenchymal markers and negative for CD45 and CD31) in samples from healthy donors. These samples were processed according to the CTCs isolation protocol and examined under a fluorescence microscope.

Whenever possible the entire sample was screened (from initial 5 mL of blood); however, in cases where there was a high number of contaminating white blood cells after CD45‐depletion, a portion of the sample was analyzed, and the number of CTCs was extrapolated proportionally (to 5 mL blood volume to allow comparison between the patients), based on the volume of the screened sample. Single cells (CTCs and up to two PBMCs, considered normal cells; NCs, presumably white blood cells – WBCs) were captured using a micromanipulator (TransferMan® 4r; Eppendorf, cat. No. 5193000012) and transferred into low‐bind 0.2 mL tubes (MAXYMumRecovery® PCR tubes; Merck, cat. No. AXYPCR02LC) containing 2 μL of lysis buffer (0.1 μL Recombinant RNase Inhibitor (Takara, cat. no. 2313A, San Jose, CA, USA) and 1.9 μL of 0.2% Triton X‐100 (Sigma Aldrich, cat. no. T9284)). The samples were then stored at −80 °C for further processing.

### 2.4 CTCs' isolation method technical performance—Spike‐in assays and enrichment factor

For technical evaluation of the CTCs isolation method, we assessed the enrichment factor, CTCs recovery rate, and immunofluorescent staining specificity. The enrichment factor was calculated as the ratio of the number of PBMCs remaining after the CTCs enrichment procedure to the initial number of PBMCs (after density gradient centrifugation). Tests were conducted using 5 mL blood samples obtained from healthy female donors, with cell counts performed using a Neubauer chamber (Marienfeld, cat. No. 0640031, Lauda‐Königshofen, Germany).

Spike‐in assays were performed in triplicate to determine the method's recovery rate (RR). MCF‐7 and MDA‐MB‐231 cells, serving as a model of eCTCs and mCTCs, respectively, were pre‐stained with Hoechst 33342 (Invitrogen, cat. No. H3570) and suspended in 1× PBS in a 96‐well plate (Falcon, cat. No. 353072, Corning, NY, USA). Cells were counted under the microscope, and 100 cells of each cell line were collected and transferred to the tubes containing blood samples from healthy donors. Following the CTCs isolation procedure, the Hoechst‐positive cells (MCF‐7 or MDA‐MB‐231) were enumerated, and the RR was calculated as the ratio of the number of cells after the complete CTCs isolation procedure to the number of spiked cells.

### 2.5 Single cells whole transcriptome preparation

Single cells were processed according to Smart‐seq2 protocol described by Picelli et al. [31]. Briefly, each sample received 1 μL of 10 μm oligo‐dT30VN primer (5′‐AAGCAGTGGTATCAACGCAGAGTACT30VN‐3′; Biomers, Ulm, Germany) and 1 μL of dNTP mix (10 mm each; Fermentas, cat. no. R0192, Waltham, MA, USA). The samples were then incubated at 72 °C for 3 min in a thermal cycler (Mastercycler X50s, Eppendorf, cat. No. 6311000010), after which they were immediately placed on ice. Following this, 5.7 μL of the reverse transcription mix was added to the samples, and reverse transcription along with preamplification (18 cycles) was conducted as outlined by Picelli et al. [31]. The preamplified samples were purified using AMpure XP beads (Beckman Coulter, cat. No. A63881, , Brea, CA, USA) in accordance with Picelli's protocol [31]. Finally, the cDNA samples were stored at −20 °C for subsequent analyses.

The quality of the single cells transcriptome was assessed by multiplex PCR amplification of three housekeeping genes: *EEF1A1* (eukaryotic translation elongation factor 1 alpha 1), *ACTB* (actin beta), and *GAPDH* (glyceraldehyde 3‐phosphate dehydrogenase), with products lengths ranging from 290 to 489 bp [32]. The multiplex PCR was prepared according to the protocol described by Durst et al. [32] and was conducted under the following conditions: an initial denaturation at 95 °C for 4 min, followed by 32 cycles of 30 s at 95 °C, 30 s at 58 °C, and 90 s at 72 °C, and a final elongation step of 7 min at 72 °C. The PCR products were separated by gel electrophoresis on a 1.5% agarose gel in 1× TAE buffer (EURX, cat. No. E0220‐02, Gdansk, Poland). The gel was run for 20 min at 100 V, followed by 30 min at 135 V. Visualization of the bands was performed using a transilluminator (Molecular Imager® Gel Doc™ XR System; Bio‐Rad, cat. No. 1070–8170, Hercules, CA, USA).

### 2.6 Gene expression analysis in single cell—qPCR and RNA‐seq

The expression of hematopoietic (*PTPRC* – protein tyrosine phosphatase receptor type C, encoding CD45), endothelial (*PECAM1*—platelet and endothelial cell adhesion molecule 1, encoding CD31), epithelial (*CDH1*—encoding ECAD, *KRT19*—encoding cytokeratin 19 (CK19), and EpCAM) and mesenchymal (*VIM*, *SERPINE1* (encoding plasminogen activator inhibitor 1—*PAI1*)) cell markers were analyzed by qPCR in single MCF‐7 and MDA‐MB‐231 cells, as well as PBMCs and CTCs. The reactions were performed in triplicate on 96‐well plates (Bio‐Rad, cat. No. MLL9601,Waltham, MA, USA) using CFX96™ Real‐Time PCR Detection System (Bio‐Rad, cat. No. 185–50‐96). Reaction mix was prepared using PowerUp™ SYBR™ Green Master Mix (Applied Biosystems, cat. No. A25741). Primers for *CDH1* (forward – TTGGAGAGACACTGCCAACTG, reverse – AGCAACTGGAGAACCATTGTCTG, product size – 151 bp), *EpCAM* (forward – GCTGGAATTGTTGTGCTGGTTA, reverse – AAGATGTCTTCGTCCCACGC, product size—189 bp), *VIM* (forward—CGAGGAGAGCAGGATTTCTC, reverse – CGTGATGCTGAGAAGTTTCGT, product size – 170 bp), *SERPINE1* (forward—CTGGTTCTGCCCAAGTTCTC, reverse – CCACTCTCGTTCACCTCGAT, product size – 179 bp), *PLS3* (forward – TTCTCCCTGGTTGGCATTGG, reverse – GGAGCTGATCGTCTTGTCCTTA, product size – 228 bp), *PTPRC* (forward – CATGGTTTCCACATTCGAGCAAT, reverse – CTTTTCTGGGGCACCAAGTG, product size – 181 bp), *PECAM1* (forward – GTCCCTGATGCCGTGGAAAG, reverse – CTCGGAACATGGATGTCCTTC, product size – 107 bp) were designed using Primer‐BLAST and checked for amplification efficiency using standard curves. Only primers with an efficiency close to 100% (±5%) were used. The reaction conditions were as follows: initial denaturation for 10 min at 95 °C, followed by 40 cycles of 15 s at 95 °C, 1 min at 60 °C, and 10 s at 95 °C. Melting curves were generated by monitoring fluorescence in the samples heated from 65 to 95 °C in 0.5 °C increments. The cycles of quantification (Cq), corrected for run‐to‐run variation, were transformed into relative quantities (RQ) according to the equation $\mathrm{RQ}=2^{{\mathrm{Cq}}_{\mathrm{MAX}}-\mathrm{Cq}}$, where Cq_MAX_ (the last cycle considered in quantitative analysis) was established as 35.

The libraries from single cells' whole transcriptomes were prepared according to Picelli et al. [31]. The concentration of cDNA from high‐quality (expressing at least 2 out of 3 housekeeping genes in the control PCR) cells from BC patients (CTCs and NCs) and BC cell lines (MCF‐7, SK‐BR‐3, MDA‐MB‐231) was analyzed using the Qubit 4 Fluorometer (Invitrogen, cat. No. Q33238) in conjunction with the Agilent High Sensitivity D5000 ScreenTape System (Invitrogen, cat. No. 5067–5592, 5067–5593, 5067–5594). Samples containing sufficient input material (1 ng, 120 samples in total) were subjected to RNA‐seq libraries preparation using the Nextera XT DNA Library Preparation Kit (Illumina, cat. No. FC‐131‐1024 and FC‐131‐1096, San Diego, CA, USA) following the protocol established by Picelli et al. [31]. cDNA fragments were ligated to index adapters (Nextera XT Index Kit, cat. No. FC‐131‐1001 or IDT® for Illumina® DNA/RNA UD Indexes Set A, cat. No. 20027213, both from Illumina) and amplified via PCR for 10 cycles. The PCR products were purified using Ampure XP beads (Beckman Coulter, cat. No. A63881) at a ratio of 0.7:1. The size distribution of the prepared libraries was verified using a 2100 Bioanalyzer Instrument (Agilent Technologies, cat. No. G2939BA, Santa Clara, CA, USA) with the Agilent High Sensitivity DNA Kit (Agilent Technologies, cat. No. 5067–4626) or the 4150 TapeStation System (Agilent Technologies, cat. No. G2992AA) with the High Sensitivity D5000 ScreenTape (Agilent Technologies, cat. No. NC1874887). If the proportion of short (135 bp) fragments exceeded 30%, the libraries were subjected to a second purification with Ampure XP beads at a ratio of 0.8:1. The libraries were then diluted to a concentration of 10 nm, pooled and supplemented with PhiX Control v3 (Illumina, cat. No. FC‐110‐3001) at a final concentration of 5% or 10%.

Single cells RNA‐seq data generated in this study have been reposited in the Gene Expression Omnibus repository (GSE268201, https://www.ncbi.nlm.nih.gov/geo/query/acc.cgi?acc=GSE268201).

### 2.7 Statistical and bioinformatics analyses

Categorical variables were tested for statistically significant differences using Pearson's *χ* ^2^ test and Fisher's exact test. Continuous data were presented using either the mean and standard deviation (SD) or the median and interquartile range (IQR), depending on the distribution of the data. Statistically significant differences in the case of quantitative variables were examined using the Kruskal–Wallis test with Dunn's multiple comparisons test and Mann–Whitney U‐test. Statistical significance was defined as *P* < 0.05. An exact *P*‐value, which considers ties among values, was computed when needed. All above‐mentioned statistical analyses were conducted using statistica software version 13.0 (StatSoft, Cracow, Poland) and GraphPad Prism software version 10.3 (GraphPad Software, San Diego, CA, USA). Corresponding figures were generated with GraphPad Prism. Survival data were compared in R using the log‐rank test; when possible, MaxCombo statistics were calculated to account for the high number of censored observations.

The best regression model explaining the relationship between the number of CTCs with different EMT phenotypes and selected clinical features (cT, cN, grade, histological type) was sought for using the multi‐response Poisson regression modeling approach. Molecular subtype was excluded from modeling due to its significant associations with the clinical characteristics ultimately included in the model. An exhaustive and iterative model selection procedure was conducted, in which all possible models (a total of 16) were systematically evaluated. Each model's performance was assessed using log‐likelihood (logLik), Akaike information criterion (AIC), and root mean squared error (RMSE), all calculated within a 10‐fold cross‐validation framework. For each model, the logLik, AIC, and RMSE values averaged across the 10 folds. To further ensure robustness and reliability, the entire 10‐fold cross‐validation procedure was repeated 500 times, with the logLik, AIC, and RMSE values being finally averaged across all iterations. The best possible model (which ultimately included cN, cT, and histological type) was identified based on the criteria of minimum average cross‐validated RMSE (avg.RMSE), maximum average log‐likelihood (avg.logLik), and minimum average AIC (avg.AIC). Models containing the grade/cN combination were not taken into consideration due to significant issues with convergence leading to abnormally high avg.RMSE values.

The model presents the IRRs with 95% confidence intervals (95% CI), calculated based on respective regression coefficients and standard errors, separately for each dependent variable. IRRs represent the multiplicative change in the expected number (count) of a particular CTCs phenotype when comparing different levels of the predictor variable to the reference category. The proportion of variability in the three dependent variables (cT, cN, histological subtype) explained by the identified optimal model was assessed using Nagelkerke pseudo‐R^2^ [33]. Individual contributions to the explained variability of the dependent variables were quantified using Nagelkerke partial pseudo‐R^2^, calculated for each predictor, separately for each dependent variable. All calculations related to Poisson modeling involving a total of 96 samples were performed in R.

In the bioinformatical analysis, sequenced reads were aligned to the GRCh38.p7 reference genome using hisat2 2.0.5 [34]. Aligned reads within the adequate GENCODE v25 gene annotation regions (https://www.gencodegenes.org/human/release_25.html) [35, 36] were counted using FeatureCounts [37]. The read counts were subsequently normalized with DESeq2 [38] in the R/Bioconductor environment [39]. Comparative analyses performed in R included fold change (FC) estimations, Student *t*‐test calculations, and false discovery rate (FDR) corrections for multiple testing, where indicated.

DEGs between two groups were identified based on the statistical significance level (*P <* 0.05, without multiple testing correction in our sample dataset and with Benjamini and Hochberg FDR correction in the validation dataset) and log_2_ fold change (log_2_(FC)). Genes with median log2(FC) ≤ −1 were considered downregulated, while genes with log_2_(FC) ≥ 1 were considered upregulated. DEGs were associated with gene ontology terms using the Functional Annotation Tool by DAVID Bioinformatics Resources 6.8 [40, 41]. The identification of red blood cells (RBCs; *n* = 138) and platelets (PLTs; *n* = 173) markers was conducted based on the list provided by Human Protein Atlas (Cluster 1: Erythroid cells – Oxygen transport and Cluster 57: Platelets – Hemostasis) [42]. These genes were removed from the DEGs analysis due to the high background level of expression.

The validation was performed on publicly available single cell RNA‐seq dataset from BC patients (GSE109761), which included 65 CTCs from BC patients and 22 NCs (white blood cells), which transcriptome was also processed via Smart‐Seq2 protocol [43]. The data was processed and normalized according to the bioinformatics pipeline described above. The EMT score of CTCs was determined according to Yu et al. by summing log_10_(RPM + 1) of mesenchymal genes (*FN1*, *CDH2*, *SERPINE1*) followed by summing log_10_(RPM + 1) of epithelial genes (*KRT5*, *KRT7*, *KRT8*, *KRT18*, *KRT19*, *EPCAM*, *CDH1*) [9]. Subsequently, the difference between the sum of the mesenchymal and epithelial genes was calculated and divided by the total number of genes (*n* = 10). EMT score quartiles were used to divide CTCs to EMT phenotypes (eCTCs < Q1, mCTCs > Q3). The gene set database used was h.all.v2024.1.Hs.symbols.gmt. GSEA was performed using the GSEA software version 4.3.3 with default parameters [44, 45], generating NES, *P*‐value and FDR.

## 3 Results

### 3.1 Method's performance in spike‐in assay

First, we assessed the specificity of the method using blood samples from six healthy volunteers. The samples were processed according to the CTC isolation protocol and examined under a fluorescence microscope for the presence of cells exhibiting eCTC, emCTC, and mCTC phenotypes. We observed dead cells (nuclear DAPI staining) with intracellular staining of mesenchymal markers (most probably VIM), distinct from surface staining of viable CTCs.

Next, we spiked 100 cells from each of the MCF‐7 and MDA‐MB‐231 cell lines into 5 mL of whole blood and performed the CTC isolation procedure. This allowed us to evaluate both the depletion rate (the ability to remove unwanted peripheral blood mononuclear cells – PBMCs) and the recovery rate (the ability to recover spiked cancer cells). The method showed a depletion rate of 98.4% of PBMCs (Fig. S2A), with a median of 7,457,059 cells at the start of the procedure compared to 86,550 cells after the procedure. The recovery rates for spiked cells showed no significant difference: 58.4% ± 6.5 (mean ± standard deviation (SD)) for epithelial MCF‐7 cells and 66.6% ± 8.5 for mesenchymal MDA‐MB‐231 cells (Fig. S2B). Representative images of the stained cells from BC cell lines are shown in Fig. S2C.

### 3.2 Inter‐ and intra‐patient heterogeneity of CTCs EMT phenotypes

**Fig. 1:**
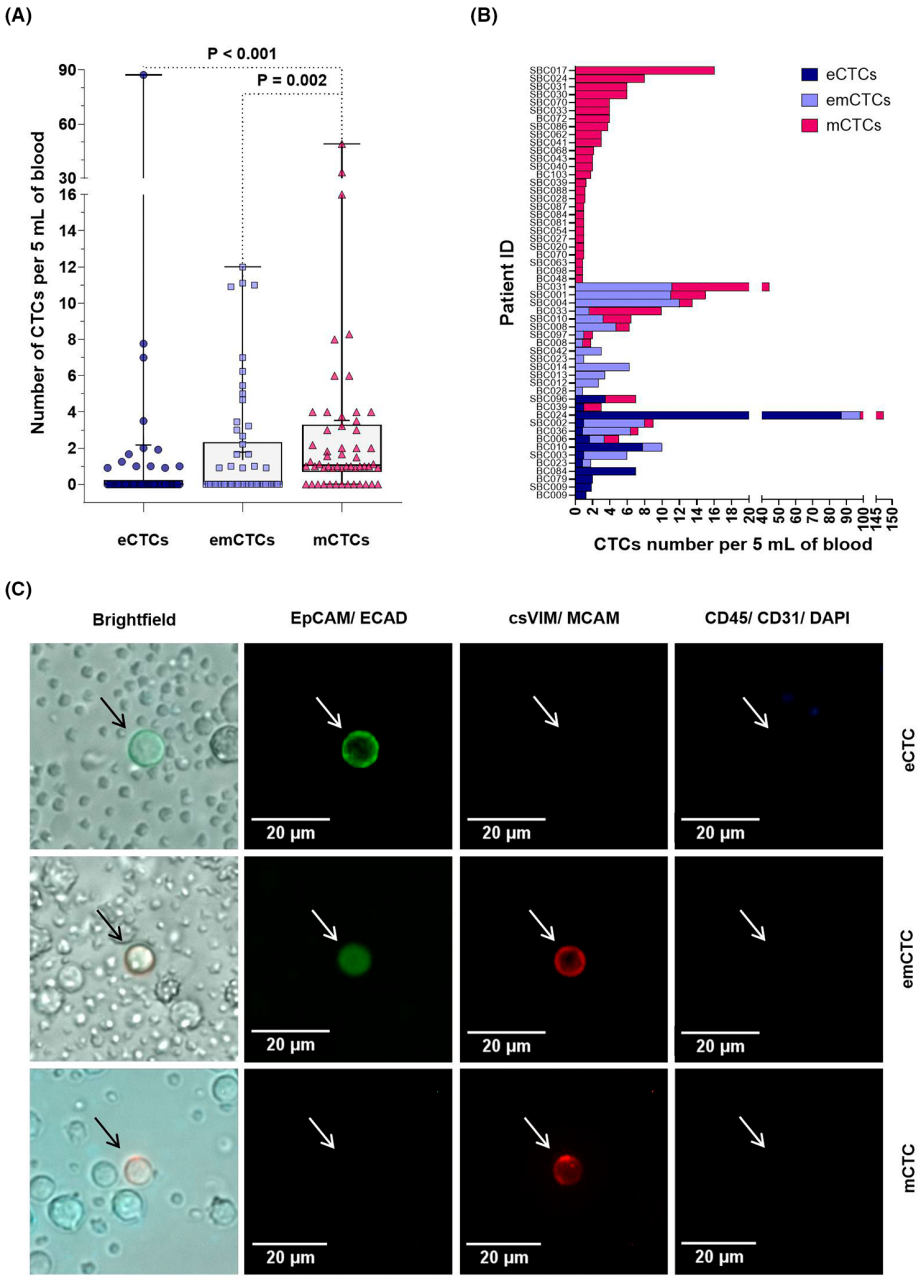
CTCs detection in patients' samples. (A) The number of CTCs by EMT phenotype (eCTCs—epithelial CTCs, emCTCs—epithelial–mesenchymal CTCs, mCTCs—mesenchymal CTCs) in non‐metastatic, untreated BC patients (*n* = 104). The box shows the interquartile range (25th to 75th percentile) with the internal line indicating the median, short line mean, and whiskers showing the range. The groups were compared using the Kruskal–Wallis test followed by Dunn's multiple comparisons test. Only significant (*P <* 0.05) differences are shown. (B) The heterogeneity of CTCs phenotypes in CTC‐positive patients (*n* = 54) shown as the absolute number of CTCs per 5 mL of blood. (C) Exemplary photos of CTCs detected in BC patients. The scale bars are shown in the corresponding photos.

Next, we evaluated the number of CTCs and their EMT phenotypes in the patients' samples. Only non‐metastatic, treatment‐naive patients were included in this analysis. Overall, 54 out of 104 (51.9%) early BC patients were CTCs‐positive, with a median of 1 CTC in a 5 mL blood sample (mean ± SD – 3.8 ± 15). The median number of eCTCs (0, IQR 0–0.2) and emCTCs (0, IQR 0–2.3) was lower than that of CTCs with purely mesenchymal phenotype (mCTCs; 1.1, IQR 0–3.3; Fig. 1A). CTC phenotypes varied among individual patients: 3.8% (*n* = 4) of the patients had only eCTCs, 26% (*n* = 27) only mCTCs, and 5.8% (*n* = 6) only emCTCs. In 16.3% (*n* = 17) of patients, two or more CTC phenotypes co‐existed (Fig. 1B, Table S2). Selected images of the detected single CTCs are shown in Fig. 1C.

### 3.3 CTCs number in patients' samples according to clinicopathological characteristics

**Fig. 2:**
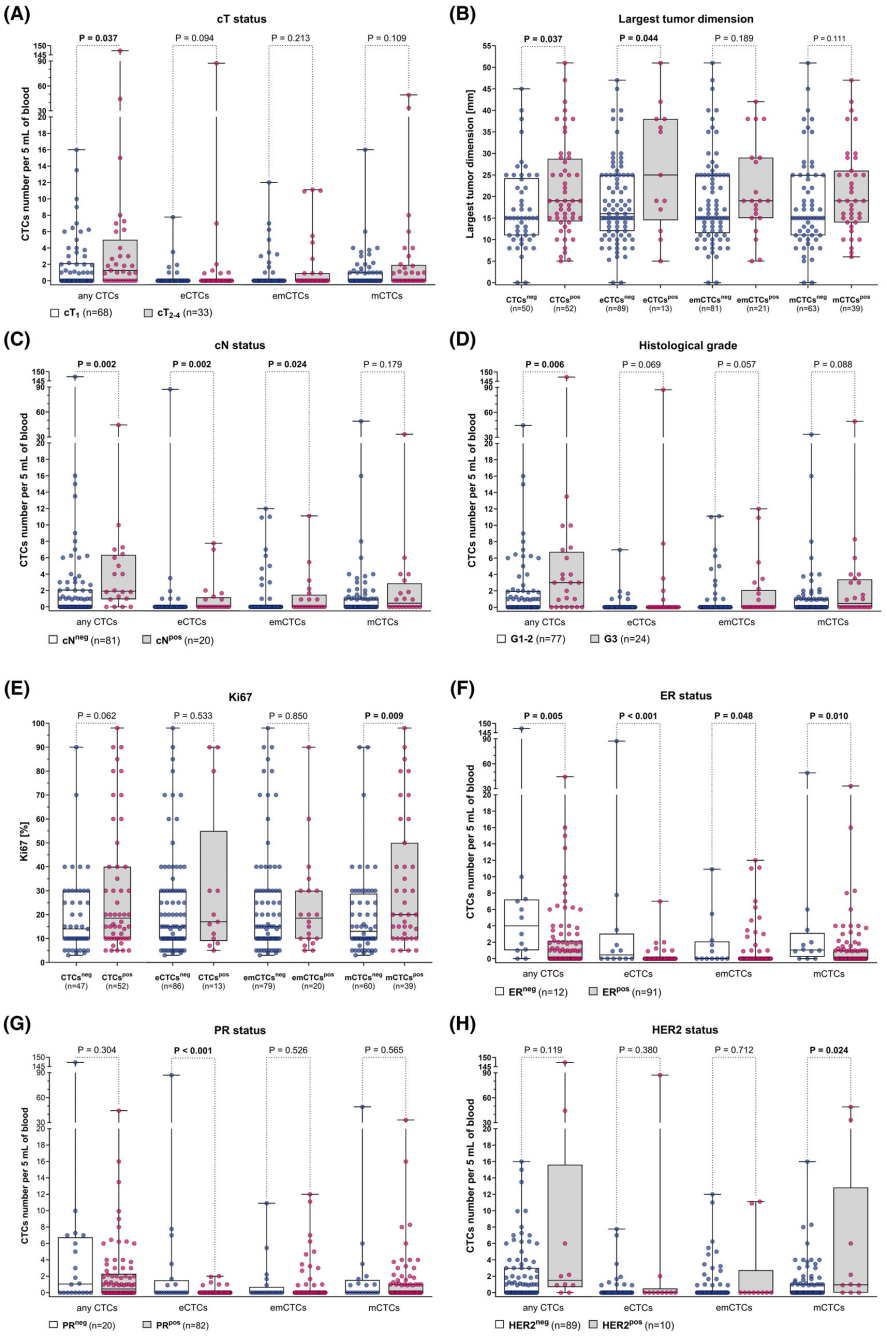
Associations between CTCs number and clinicopathological parameters in early non‐treated BC patients (*n* = 104). The number of any CTCs and CTCs divided by their EMT phenotype (eCTCs, epithelial CTCs; emCTCs, epithelial–mesenchymal CTCs; mCTCs, mesenchymal CTCs) analyzed according to (A) cT, (B) largest tumor dimension, (C) cN, (D) tumor grade, (E) percent of proliferating (Ki67‐positive) cells in tumor, (F) ER status, (G) PR status, and (H) HER2 status according to the presence of any CTCs or CTCs with specific phenotypes. The box shows interquartile range (25th to 75th percentile) with the internal line indicating the median and short line mean. The groups were compared using the Mann–Whitney U‐test.

The median number of total CTCs (all EMT phenotypes) was higher in patients with larger tumors – 1.3 CTCs in cT_2‐4_ (IQR 0–5) vs 0 (IQR 0–2.1) CTCs in cT_1_ (*P* = 0.037; Fig. 2A, Table S3). Moreover, CTC‐positive patients (considering all CTCs phenotypes) and eCTCs‐positive patients exhibited a greater largest tumor dimension in comparison to patients negative for CTCs or patients negative for eCTCs, with medians of 15 vs 19 mm (for all CTCs, *P =* 0.037) and 16 vs 25 mm (for eCTCs, *P =* 0.044; Fig. 2B). Median counts of total CTCs, as well as eCTCs and emCTCs, were elevated in lymph node‐positive (cN^pos^) vs lymph node‐negative patients (cN^neg^) – 1.9 (IQR 0.9–6.3) vs 0 (IQR 0–2.1) for total CTCs (*P =* 0.002), 0 (IQR 0–1.2) vs 0 (IQR 0–0) for eCTCs (*P* = 0.002), and 0 (IQR 0–1.5) vs 0 (IQR 0–0) for emCTCs (*P* = 0.024; Fig. 2C, Table S3). Additionally, the median number of total CTCs was significantly higher in less differentiated (G3) than in G1‐2 tumors – 3 (IQR 0–6.8) vs 0 (IQR 0–2) CTCs (*P =* 0.006; Fig. 2D, Table S3). The median percentage of proliferating (Ki67‐positive) cancer cells in the primary tumors was higher in mCTC‐positive patients than in mCTC‐negative patients (20% vs 13%, *P =* 0.009; Fig. 2E). Regarding hormone receptor status, patients with estrogen receptor (ER)‐negative (ER^neg^) tumors had a significantly higher median number of total CTCs compared to those with ER‐positive (ER^pos^) tumors – 4 (IQR 1–7.2) vs 0 (IQR 0–2.2) CTCs (*P =* 0.005); this was also observed for other CTCs subtypes – 0.5 (IQR 0–3) eCTCs in ER^neg^ vs 0 (IQR 0–0) in ER^pos^ (*P* < 0.001), 0 (IQR 0–2.1) emCTCs in ER^neg^ vs 0 (IQR 0–0) in ER^pos^ (*P =* 0.048), 1 (IQR 0.2–3.1) mCTCs in ER^neg^ vs 0 (IQR 0–1) in ER^pos^ (*P =* 0.01; Fig. 2F, Table S3). Similarly, patients with progesterone receptor (PR)‐negative (PR^neg^) tumors exhibited a higher median number of eCTCs – 0 (IQR 0–1.5) eCTCs in PR^neg^ vs 0 (IQR 0–0) eCTCs in PR^pos^ (*P* < 0.001; Fig. 2G, Table S3). Conversely, patients with human epidermal growth factor receptor type 2 (HER2)‐positive tumors had a higher median number of mCTCs compared to HER2‐negative cases – 1 (IQR 0–12.8) vs 0 (IQR 0–1.1) mCTCs (*P =* 0.024; Fig. 2H, Table S3). When analyzing associations between CTCs‐positivity (as nominal value) and HER2 status, patients with HER2‐positive tumors were more often mCTCs‐positive (70% vs 35%, *P =* 0.04; Table S4).

Co‐occurrence of CTCs with different EMT phenotypes was more frequent in TNBC than in luminal tumor subtypes. Specifically, 86% of TNBC patients had CTCs with at least two different EMT phenotypes, compared to only 17% of patients with luminal A tumors (*P* = 0.007; Fig. S3A). Similar differences in the heterogeneity of CTCs phenotypes were observed for ER and PR receptors assessed separately (Fig. S3B). Overall, the presence of CTCs with various phenotypes is associated with characteristics of more aggressive disease.

In the survival analysis, six deaths were observed during the median follow‐up of 4.6 years. Given the small number of events and the frequent co‐occurrence of different CTCs phenotypes within individual patients, those with mesenchymal features (emCTCs and mCTCs) were analyzed as one group; also, CTCs‐negative and eCTCs‐only positive patients were combined. Five out of six deaths occurred in patients with detectable emCTCs or mCTCs, and one in a patient without detectable CTCs (*P* = 0.07, log‐rank test; Fig. S4A). When correcting for a high degree of censored data using MaxCombo test statistics, the results were not significant (*P* = 0.104). Similarly, analysis of three groups (CTCs‐negative, eCTCs‐only positive, emCTC or mCTCs‐positive) showed a non‐significant difference in survival (*P* = 0.19, log‐rank test; MaxCombo test could not be calculated; Fig. S4B). Extending the follow‐up period could increase event numbers and enhance the power to detect survival differences.

#### 3.3.1 Regression analysis

The best‐fitting multi‐response Poisson regression model (Table S5), which included cT, cN, and histological type, explained around 81% of the variability of the number of all three CTCs phenotypes (eCTCs, emCTCs, mCTCs) and was highly statistically significant (*P* < 0.0001). It revealed that lymph node involvement (cN) was the strongest predictor, with the presence of any metastases in regional lymph nodes (cN^pos^) being characterized by significantly increased numbers of CTCs across all phenotypes: (eCTCs: incidence rate ratio – IRR = 14.1, *P* < 0.001; emCTCs – IRR = 2.37, *P* = 0.002; mCTCs – IRR = 2.85, *P* < 0.001; Table 1). Larger tumors (cT_2–4_) were found to be associated with a significantly increased number of mCTCs (IRR = 2.2, *P* < 0.001), while the lobular type of cancer was associated with a reduced number of emCTCs (IRR = 0.08, *P* = 0.012) and mCTCs (IRR = 0.34, *P* < 0.005; Table 1).

**Table 1:** Results of multi‐response regression modeling. IRRs with 95% CI for the best‐fitting multi‐response Poisson regression model are presented. The model includes clinical predictors (cT, cN, histological type). Three dependent variables were modeled simultaneously: the number of eCTCs, emCTCs, and mCTCs.

Predictor	Incidence rate ratio [95% CI]					
	eCTCs	*P*	emCTCs	*P*	mCTCs	*P*
cT_234_ vs cT_1_	0.82 [0.37–1.81]	0.62	1.57 [0.91–2.7]	0.103	2.22 [1.49–3.31]	<0.001
cN^pos^ vs cN^neg^	14.12 [5.78–34.49]	<0.001	2.37 [1.37–4.08]	0.002	2.85 [1.92–4.22]	<0.001
Lobular vs ductal	0 [0‐Inf]	0.97	0.08 [0.01–0.57]	0.012	0.34 [0.17–0.68]	0.002

Overall, cN was the primary contributor to the variability in the number of eCTCs, explaining almost 38% of the variability (*P* < 0.001), while cT and histological type contributed only insignificantly (Table 2). In the case of emCTCs, histological type explained the largest part of the emCTCs number variability (over 14%, *P* = 0.012), followed by cN (almost 8%, *P* = 0.002), with cT contributing insignificantly. In mCTCs, cN explained almost 16% (*P* < 0.001) of the number variability, cT and histological type contributing an additional 8.57% (*P* < 0.001) and 6.75% (*P* = 0.002), respectively (Table 2).

**Table 2:** Proportion of variability in the number of CTCs explained by clinical predictors in the regression model. The percentage of variability in the number of CTCs with different EMT phenotypes (eCTCs, emCTCs, mCTCs) explained by individual clinical predictors (cN, cT, histological subtype) in the best‐fitting Poisson regression model are shown. Higher percentages indicate a greater contribution of the predictor to the variability of the number of respective CTCs.

Predictor	Variability explained [%]					
	eCTCs	*P*	emCTCs	*P*	mCTCs	*P*
cT	0.19	0.62	2.17	0.103	8.57	<0.001
cN	37.86	<0.001	7.89	0.002	15.94	<0.001
Histological subtype	7.48	0.97	14.32	0.012	6.75	0.002

### 3.4 Transcriptomic profiling of single cells

#### 3.4.1 Cell lines

To evaluate the impact of the CTCs enrichment method on the transcriptome quality of cancer cells, single MCF‐7 and MDA‐MB‐231 cells were collected by micromanipulation at two distinct time points: (1) immediately after trypsinization, representing single cells with optimal transcriptome quality, and (2) following spiking into a blood sample and the subsequent CTCs isolation procedure. The results revealed that the transcriptome quality of both cell lines remained largely unaffected by the CTCs isolation process. Specifically, 100% of MDA‐MB‐231 and 89% of MCF‐7 cells exhibited good‐quality transcriptomes, defined as the expression of at least two out of three housekeeping genes in multiplex PCR (Fig. S5A). Furthermore, qPCR analysis of five EMT marker genes (cadherin 1 – *CDH1*, *EpCAM*, *VIM*, *plastin3 – PLS3*, serine proteinase inhibitor family E member 1 – *SERPINE1*) enabled the differentiation of epithelial and mesenchymal phenotypes in single MCF‐7 and MDA‐MB‐231 cells, as well as allowing for their separation from blood cells (normal cells – NCs) based on CD45 (hematopoietic cell marker) expression (Fig. S5B).

#### 3.4.2 Patient's samples

However, the quality of the transcriptome of patient‐derived cells was worse than that of the cell lines. Out of 104 CTCs and 65 NCs isolated from 46 patients, 51 CTCs (49%) and 39 NCs (60%) had good‐quality transcriptomes (at least two out of three bands in control PCR, Fig. S5C). Especially, the transcriptome of emCTCs showed decreased quality in comparison to eCTCs, mCTCs, and NCs – only 22.6% of emCTCs had two or more bands in control PCR. A clear discrepancy was observed in the percentage of high‐quality cells from patients' samples (Fig. S5C) compared to the model system (spiked‐in cell lines– Fig. S5A); this could be attributed to the stressful conditions that CTCs face in the bloodstream. While phenotyping based on gene expression using qPCR was possible for cells from BC cell lines (Fig. S5B), it was not possible to definitively classify CTCs from clinical samples to EMT phenotypes (Fig. S5D); no mRNA expression of mesenchymal marker genes (*SERPINE1*, *PLS3*) was detected in CTCs (data not shown), which were used to confirm the phenotypes of BC cell lines (Fig. S5B). Nevertheless, emCTCs exhibited significantly higher levels of epithelial marker gene expression compared to eCTCs and mCTCs for *CDH1* (median in emCTCs – 2.2 vs eCTCs and mCTCs – 0, *P* < 0.001 for both) and *EpCAM* (median in emCTCs – 0.8 vs eCTCs and mCTCs – 0, *P* < 0.001 for both; Fig. S5D). Since gene expression by qPCR detects only selected regions of a transcript, predetermined by primer design, it is still possible that a gene is expressed, but the given fragment of the transcript was absent (e.g., degraded or not efficiently reverse‐transcribed). Therefore, applying RNA‐Seq for transcriptomic characterization would allow for broader profiling, as in RNA‐Seq different fragments of any given transcript can be sequenced. For detailed bioinformatics analysis, only cells (46/48) with good‐quality RNA‐Seq results (defined as expression of at least 800 mapped genes per cell) were included; one CTC and one NC were removed from the analysis (Fig. S6A).

**Fig. 3:**
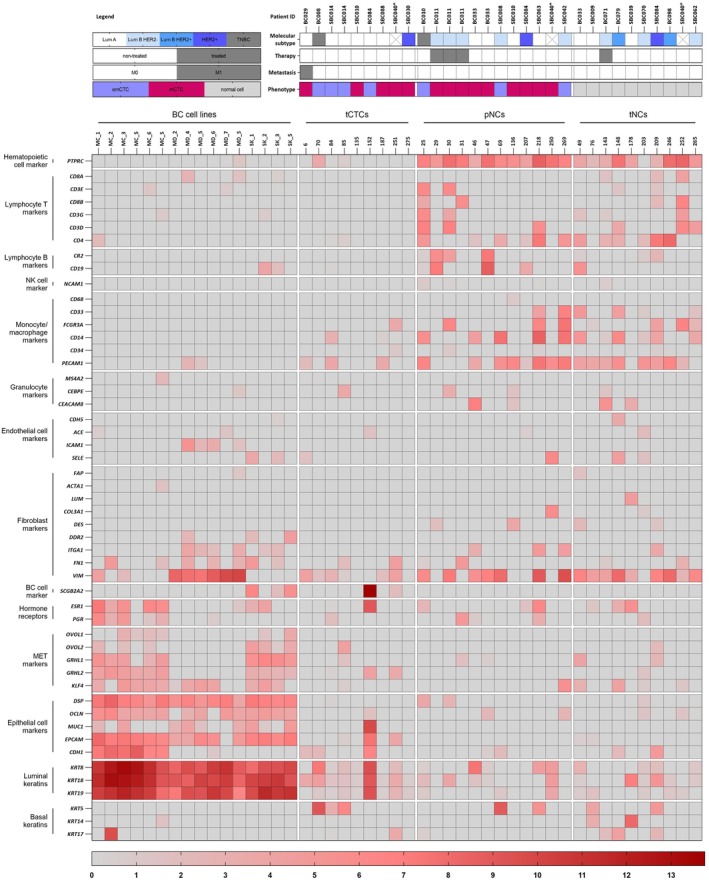
Gene expression profile of BC markers and hematopoietic cell lineages markers (rows) in single cells (columns presents cells' ID). Single cells from BC cell lines were picked directly after trypsinization (*n* = 14; MC_1, _2, _3, _5, _6 – MCF‐7, MD_2, _4, _5, _6, _7 – MDA‐MB‐231, SK_1, _2, _3, _5 – SK‐BR‐3) and after spike‐in test (*n* = 2; MC_S, MD_S). Single cells from BC patients are divided into true CTCs (tCTCs; *n* = 9), potential NCs (pNCs; *n* = 12), and true normal cells (tNCs; *n* = 10). The legend above tCTCs, pNCs, and tNCs refers to BC patients from which the given cells were picked. *The exact molecular subtype for patient SBC040 (ER ≥1%, PR ≥20%, Ki67 > 20%) could not be determined due to missing HER2 status.

The expression profile of BC marker genes and hematopoietic cell lineage marker genes in high‐quality transcriptome cells indicates that some CTCs express immune cell genes (Fig. 3). This raises doubts about their neoplastic origin, particularly in cases where multiple different immune cell marker genes are simultaneously observed (e.g., cell 25, 30). For this reason, we used the information from the protein (EpCAM/ECAD/csVIM/MCAM/CD45/CD31 detected during immunofluorescent staining of cells before micromanipulatory isolation) and gene expression (RNA‐Seq detecting cancer and immune cell markers) to classify cells into three groups with expression profiles progressively indicating characteristics of immune system cells:

1. True CTCs (tCTCs) – cells picked based on positive staining of epithelial and/or mesenchymal cancer markers and the absence of signal from CD45/CD31 at the protein level. Transcriptomically, these cells do not express (gene expression level <4) immune system cell markers.
2. Potential normal cells (pNCs) – cells picked based on positive staining of epithelial and/or mesenchymal markers and the absence of signal from CD45/CD31 at the protein level. Transcriptomically, these cells exhibit expression (≥4) of the immune system and/or endothelial cell markers.
3. True normal cells (tNCs) – cells picked based on the absence of signal from epithelial and mesenchymal markers at the protein level.

The characteristics of the patients from which the single cells were picked are shown in Table S6.

Single cells from BC cell lines exhibited strong expression of luminal keratins, epithelial markers, and some of mesenchymal‐epithelial transition (MET) factors. tCTCs showed low‐level expression of luminal keratins, while some cells expressed basal keratins 5 and 17, or mammary transcript mammaglobin 1 (encoded by the *SCGB2A2* gene; Fig. 3).

**Fig. 4:**
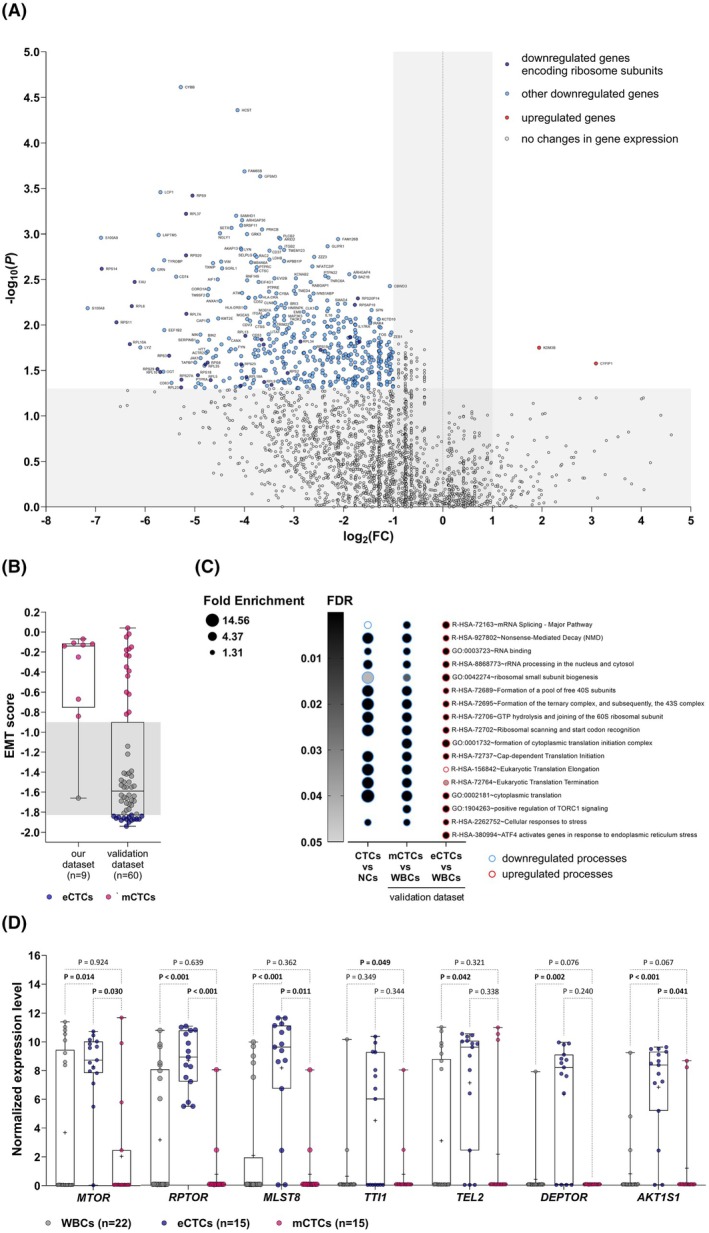
Transcriptomic differences between CTCs and white blood/normal cells. (A) Differentially expressed genes between tCTCs (true CTCs; *n* = 9) and NCs (normal cells of the PBMC fraction; *n* = 10)—downregulated (log_2_(FC) ≤ −1) genes encoding ribosome subunits are shown in dark blue, other downregulated genes – in light blue, upregulated (log_2_(FC) ≥ 1) genes – in red. The genes of unchanged expression are shown in gray. (B) EMT score of CTCs from our and validation datasets. The box shows interquartile range (25th to 75th percentile) with an internal line indicating median and “+” sign indicating mean, while whiskers show range. The groups were compared using the Mann–Whitney U‐test. (C) Most significantly changed processes in CTCs in our (no FDR correction) and validation datasets (with FDR). (D) Normalized gene expression level of mTORC1 complex components in epithelial CTCs (eCTCs; *n* = 15), mesenchymal CTCs (mCTCs; *n* = 15), and white blood cells (WBC; *n* = 22) from the validation set. The box extends from the 25th to 75th percentiles with an internal line indicating median and “+” sign indicating mean, while whiskers show range. The groups were compared using the Kruskal–Wallis test followed by Dunn's multiple comparisons test. eCTCs, epithelial CTCs; emCTCs, epithelial–mesenchymal CTCs; mCTCs, mesenchymal CTCs.

Analysis of differentially expressed genes (DEGs) between tCTCs and tNCs revealed that 471 genes were downregulated in tCTCs compared to tNCs, while only two genes showed increased expression (Fig. 4A; detailed gene list is available in Table S7). Many of the downregulated genes encode ribosomal proteins (RPs), such as *RPL15*, *RPL6*, *RPS11*, and *RPS14*, previously described to be downregulated in CTCs of mesenchymal phenotype [46]. Interestingly, the most significantly upregulated gene in tCTCs was *CYFIP1* (cytoplasmic FMR1 interacting protein 1), which acts as a translation inhibitor [47]. The next upregulated gene in CTCs was *KDM3B* (lysine demethylase 3B), an iron‐binding lysine demethylase targeting residues on histone tails [48], described to activate mTORC1 signaling – an important pro‐survival pathway [49]. The most altered processes in which the downregulated genes are involved were connected to transcription and translation (Fig. 4B; full list of the processes is presented in Table S8). The disturbance of these fundamental genes' expression would explain the overall decreased number of genes expressed in CTCs compared to NCs (Fig. S6B); while the number of assigned transcripts was similar in tCTCs and tNCs (Fig. S6C), the number of genes detected in tCTCs was lower than in tNCs (*P =* 0.041, median 3332 vs 5249; Fig. S6B). This finding suggests that the complexity of the CTCs transcriptome might be lower than that of non‐cancerous NCs. Other processes downregulated in CTCs were characteristic of immune cells, further confirming the non‐leukocyte nature of the captured CTCs (Table S8).

We have validated our observations in an independent dataset of BC CTCs (*n* = 65) and WBCs (*n* = 22; GSE109761). EMT scores of CTCs from our dataset were comparable to mCTCs from the validation dataset (Fig. 4B). DEGs and GO analysis confirmed that in mCTCs (in comparison to WBCs), translation, RNA binding, and mRNA splicing are diminished (Fig. 4C, Tables S9 and S10). Conversely, eCTCs (vs WBCs) demonstrated upregulation of these processes, suggesting that epithelial and mesenchymal CTCs differentially regulate gene expression and translation (Fig. 4C, Tables S11 and S12). The expression of core ribosomal proteins in CTCs also differs depending on EMT status and is significantly lower in mCTCs vs eCTCs (Fig. S7, Table S13). Among ontologies differentially activated in eCTCs and mCTCs were mTORC1 and ATF4 (activating transcription factor 4) (Fig. 4C); gene set enrichment analysis (GSEA) also indicated increased mTORC1 signaling in eCTCs (vs WBCs; NES = 1.43, *P* = 0.002; Fig. S8A), and its decrease in mCTCs (NES = −1.33, *P* = 0.053; Fig. S8B). Genes of the mTORC1 complex, including the *MTOR* (mammalian target of rapamycin) catalytical subunit and core proteins – *RPTOR* (regulatory‐associated protein of *MTOR*) and *MLST8* (*MTOR*‐associated protein, LST8 homolog) had increased expression in eCTCs, significantly higher than in mCTCs and WBCs (Fig. 4D). Similarly, *ATF4* was increased in eCTCs, but not in mCTCs, which might suggest a coordinated response promoting protein synthesis in eCTCs and not in mCTCs (Fig. S8C). Finally, increased expression of *KDM3B* (identified in our set; Fig. 4A) was observed in both eCTCs and mCTCs in the validation set (Fig. S8C).

## 4 Discussion

Understanding the mechanism of metastatic dissemination is the key point for intercepting cancer spread. During metastatic colonization, cells need to invariably adapt to survive in the very different niches in which they naturally reside. In particular, the process of EMT is intensely studied in the context of making cancer cells more resilient to changing environments. Effects of EMT are pleiotropic, often context‐specific, e.g., depending on the location of cancer cells (primary tumor vs CTCs). It is in CTCs where the activation of EMT was strongly associated with tumor progression and resistance to treatment [9]. Despite the agreement on the importance of better characterization of EMT features in CTCs, existing methods of CTC capture and characterization are still limited. Though a large spectrum of methods is being developed to allow identification of mCTCs, many of these new techniques have not been validated in patients' samples [50]. Rarely, methods also allow obtaining living CTCs, which can be transcriptomically characterized to assess operating biological processes. This is especially true for non‐metastatic patients, where CTCs are rare [50].

In the current work, we have described a method allowing recovery of BC CTCs with different EMT phenotypes using a mixture of epithelial (EpCAM, ECAD) and mesenchymal (csVIM, MCAM) markers analyzed by immunofluorescence that could be further transcriptomically characterized. The developed method did not preferentially isolate cells with a given EMT phenotype; cells with epithelial and mesenchymal phenotypes were isolated with equal efficiency, while simultaneously obtaining a higher recovery rate of the mesenchymal CTCs than CellSearch® (66.6% vs 23.7%) [50]. The method was implemented for the evaluation of the number and phenotype of CTCs in a group of mostly non‐metastatic BC patients.

We observed that a large fraction of patients (48%) had CTCs exhibiting mesenchymal features (emCTCs or mCTCs phenotype), with fully mCTCs being the most common (25.9%). This phenotype was more frequently observed in proliferative, ER‐negative, or HER2‐positive tumors, indicating the link between EMT in CTCs and worse clinicopathological characteristics of the patients. Although not statistically significant, the observed trend suggests poorer survival in patients with detectable mCTCs and emCTCs. Given the limited number of events and high censoring rate, these results warrant extended follow‐up for confirmation. Because different CTC phenotypes often co‐occurred within the same patient, it was not possible to assess the impact of individual phenotypes on survival due to the limited number of events. The percentage of mCTCs‐positive patients was slightly higher than reported in the literature—maximal 30% [50], but markers used in our analysis—csVIM and MCAM, have been shown to significantly improve the detection rate of CTCs lacking EpCAM expression [8, 21, 22]. In non‐metastatic patients, EMT transcription factors (TWIST1, SNAIL, SLUG) have been predominantly used to detect mCTCs; however, their sensitivity is relatively low [50], which could result in smaller mCTCs positivity rates in other studies. Percentages of patients with CTCs of epithelial features (eCTCs and/or emCTCs – 25.9%) were similar to those in other studies (median 22.3%, range 10–45%) [51, 52, 53, 54, 55, 56, 57, 58, 59, 60, 61]. eCTCs were found in patients with larger, ER‐negative, and PR‐negative tumors and with involved lymph nodes. It shows that detecting CTCs across the full spectrum of EMT phenotypes may carry important clinical information. Additionally, CTCs‐positive TNBC patients demonstrated greater phenotypic heterogeneity in their CTCs compared to those with luminal A BC subtypes (86% vs 17% of patients presenting with more than one CTC phenotype in a blood sample). Since TNBC patients often exhibit poorer clinicopathological characteristics and worse prognoses [5], the presence of CTCs with diverse EMT phenotypes within a single patient may reflect increased plasticity of this molecular subtype of BC and could be associated with its heightened aggressiveness.

In the Poisson regression model of CTCs dissemination, lobular histological subtype was found to be linked with decreased levels of emCTCs and mCTCs. Lymph node involvement (cN) was the most important predictor of the presence of all CTC phenotypes. On the other hand, the model suggests that with increasing tumor size, dissemination of mCTCs, but not eCTCs and emCTCs, increases. Nevertheless, it still needs to be kept in mind that the Poisson model may suffer from limitations associated with the Hauck‐Donner effect, potentially arising from the reduced sample size, which could have led to near‐perfect separation or sparse data in certain predictor combinations. As a result, careful interpretation of the calculated IRRs is warranted, as some estimates may exhibit limited reliability due to these underlying issues.

In advancing the molecular characterization of CTCs, the developed method did not compromise the transcriptome quality of cells during spike‐in tests. However, the proportion of high‐quality cells obtained from BC patients was significantly lower compared to that from the model system using cell lines. This points to a limitation of using well‐established cancer cell lines as model systems in CTCs research. CTCs are subjected to various stressors, including mechanical damage, oxidative stress, immune system surveillance, and a lack of physiological signals [62]. These factors can lead to the suppression of gene expression and/or the degradation of mRNA, which is inherently unstable even under homeostatic conditions. This instability further restricts the ability to phenotype CTCs using low‐throughput methods, such as qPCR, prompting high‐throughput profiling like RNA‐Seq. In our study, transcriptomic profiles of tCTCs (not expressing blood cell markers) vs tNCs were compared using RNA‐Seq; we observed strong downregulation of ribosomal proteins and factors involved in the translation process, which was also confirmed in the CTCs validation set. A decrease in translation during EMT is consistent with the results of Ebright et al. showing that mCTCs of BC patients have lower levels of ribosomal proteins than eCTCs [46]. Identified ontologies – increased mTORC1 and ATF4 signaling in eCTCs, could potentially orchestrate this actively translating phenotype; mTORC1 is a main anabolic pathway driving protein synthesis [63], whereas ATF4 might indicate an adaptive response aiming at alleviation of cellular stress [64]. However, the role of mTORC1 and ATF4 in cancer is far from being understood [65, 66], and further investigations of their role in cancer metastasis in the context of EMT are required.

We also observed upregulation of *KDM3B* in our CTCs cohort and in the validation set. KDM3B, being a histone demethylase, was recently described to activate mTORC1 signaling via increased expression of its essential subunit—*RPTOR* [67]. Additionally, *KDM3B* expression was found vital for the development of the aggressive phenotype in castration‐resistant prostate cancer [68]; it was associated with an increased proportion of cells with a stem cell phenotype, decreased apoptosis, and chemoresistance in colorectal cancer models [69]. KDM3B overexpression has been documented in epithelial tumors [70, 71, 72, 73, 74], where it correlates with unfavorable clinical‐pathological characteristics and poorer prognosis [72, 74].

Considering therapeutic implications, several drugs used in BC treatment have a mode of action that operates via inhibition of transcription and translation processes [75, 76, 77]. The most relevant in the context of our findings are inhibitors of mTOR, such as everolimus, which primarily inhibits the mTORC1 complex [78]. However, data from the BC mouse model suggest that mTOR inhibition might induce a stem cell phenotype in cancer cells, and in the experimental metastasis model, increase the number of lung metastases [79]. To overcome this effect, the inhibitor of integrated stress response (ISRIB) was effective in eliminating chemoresistant cancer cells [79]. Also, KDM3B inhibitors are undergoing intensive investigation for their potential application in cancer treatment, including BC [80, 81, 82]. However, it is essential to recognize that these inhibitors remain in the preclinical research phase, and their efficacy and safety must be thoroughly evaluated before they can be integrated into clinical practice.

## 5 Conclusion

Our analysis showed that all EMT phenotypes of CTCs are more prevalent in early BC patients with lymph node involvement, while selected CTC phenotypes were increased in ER‐negative and HER2‐positive patients. We identified global changes in translation processes, possibly regulated via mTORC1 – a decrease in mCTCs and an increase in eCTCs.

## Conflict of interest

The authors declare that they have no conflict of interest.

## Author contributions

Conceptualization: AM; data curation: JT, TS, AS, AgS, AM; formal analysis: JT, TS, AS, PMG, AM; funding acquisition: AJŻ, AM; investigation: JT, JR, ŁA, AM; methodology: JT, AM; resources: GS, ES, MS, MC, BT, WK, AJŻ, AM; supervision: AJŻ, AM; visualization: JT; writing – original draft: JT, AM; writing – review and editing: JT, JR, TS, AS, MS, MC, PMG, BT, ŁA, AgS, GS, ES, WK, AJŻ, AM.

## Supporting information


**Fig. S1.** Workflow for the detection and isolation of single CTCs for subsequent transcriptomic profiling.
**Fig. S2.** Technical performance of CTCs' enrichment method.
**Fig. S3.** (A) CTCs phenotypic heterogeneity according to molecular subtype and (B) clinicopathological characteristics of BC patients.
**Fig. S4.** Survival probability of patients depending on presence and phenotype of detected CTCs.
**Fig. S5.** Transcriptomic characterization of single cells.
**Fig. S6.** Quality of patients' CTCs and NCs transcriptome.
**Fig. S7.** Gene expression profile of core ribosomal subunits (rows) in single CTCs and WBCs from validation dataset.
**Fig. S8.** Gene expression in the validation set.
**Table S1.** Clinicopathological characteristics of early treatment‐naïve BC patients (*n* = 104).
**Table S2.** The number and percentage of patients with CTCs with given EMT phenotypes.
**Table S3.** The number of CTCs with a given EMT phenotypes in correlation to patients' clinicopathological characteristics.
**Table S4.** Correlation between clinicopathological patients' characteristics and presence of CTCs with different EMT phenotypes.
**Table S5.** Summary of the regression model selection procedure.
**Table S6.** The characteristics of the patients from whom single cells were picked.

**Table S7.** Differentially expressed genes in true CTCs (tCTCs) vs true normal cells of the PBMC fraction (tNCs).
**Table S8.** The processes disturbed in true CTCs (tCTCs) vs true normal cells of the PBMC fraction (tNCs).
**Table S9.** Differentially expressed genes in mesenchymal CTCs (mCTCs) vs white blood cells (WBCs) in the validation set.
**Table S10.** The processes inhibited in mesenchymal CTCs (mCTCs) vs white blood cells (WBCs) in the validation set.
**Table S11.** Differentially expressed genes in epithelial CTCs (eCTCs) vs white blood cells (WBCs) in the validation set.
**Table S12.** The processes changed in epithelial CTCs (eCTCs) vs white blood cells (WBCs) in the validation set.
**Table S13.** Differences in expression of genes encoding large and small core ribosomal subunits.

## Data Availability

The datasets generated and analyzed during the current study are available in the Gene Expression Omnibus repository; https://www.ncbi.nlm.nih.gov/geo/query/acc.cgi?acc=GSE268201.
