# Supplementary material for: Characterizing epithelial‐mesenchymal transition‐linked heterogeneity in breast cancer circulating tumor cells at a single‐cell level
Source: Mol Oncol. 2025 Oct 5;19(12):3685–705. doi: 10.1002/1878-0261.70132 (PMC12688172; doi:10.1002/1878-0261.70132)
Supplement: Supplementary file 1 — Fig. S1. Workflow for the detection and isolation of single CTCs for subsequent transcriptomic profiling. Fig. S2. Technical performance of CTCs' enrichment method. Fig. S3. (A) CTCs phenotypic heterogeneity according to molecular subtype and (B) clinicopathological characteristics of BC patients. Fig. S4. Survival probability of patients depending on presence and phenotype of detected CTCs. Fig. S5. Transcriptomic characterization of single cells. Fig. S6. Quality of patients' CTCs and NCs transcriptome. Fig. S7. Gene expression profile of core ribosomal subunits (rows) in single CTCs and WBCs from validation dataset. Fig. S8. Gene expression in the validation set. Table S1. Clinicopathological characteristics of early treatment‐naïve BC patients (n = 104). Table S2. The number and percentage of patients with CTCs with given EMT phenotypes. Table S3. The number of CTCs with a given EMT phenotypes in correlation to patients' clinicopathological characteristics. Table S4. Correlation between clinicopathological patients' characteristics and presence of CTCs with different EMT phenotypes. Table S5. Summary of the regression model selection procedure. Table S6. The characteristics of the patients from whom single cells were picked. [file MOL2-19-3685-s001.docx]

**
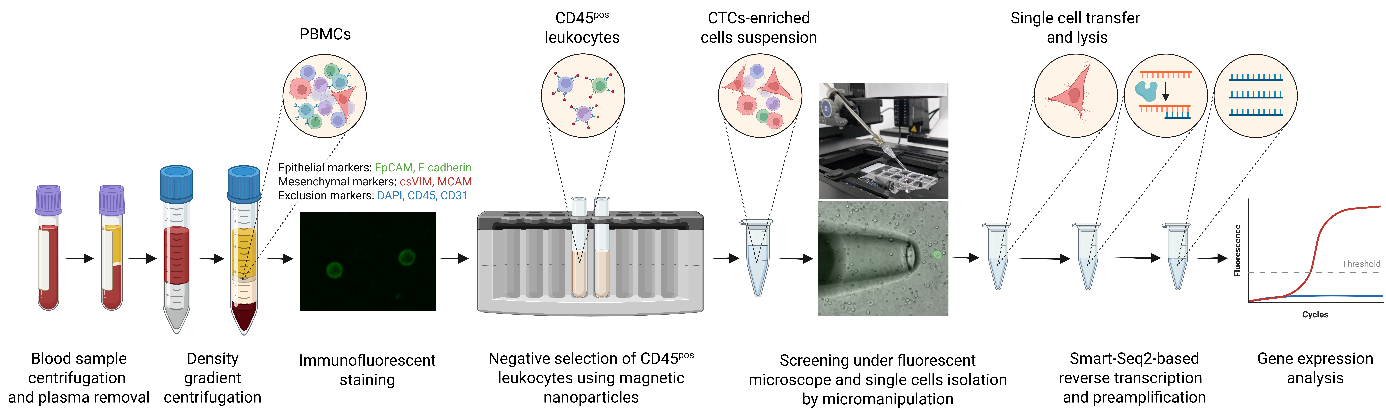
**

**Figure S1** **Workflow for the detection and isolation of single CTCs for subsequent transcriptomic profiling.** Based on a figure originally published in Topa J, Żaczek AJ, Markiewicz A. Isolation of Viable Epithelial and Mesenchymal Circulating Tumor Cells from Breast Cancer Patients. Methods Mol Biol. 2024;2752:43-52. doi: 10.1007/978-1-0716-3621-3_3)**.**

**
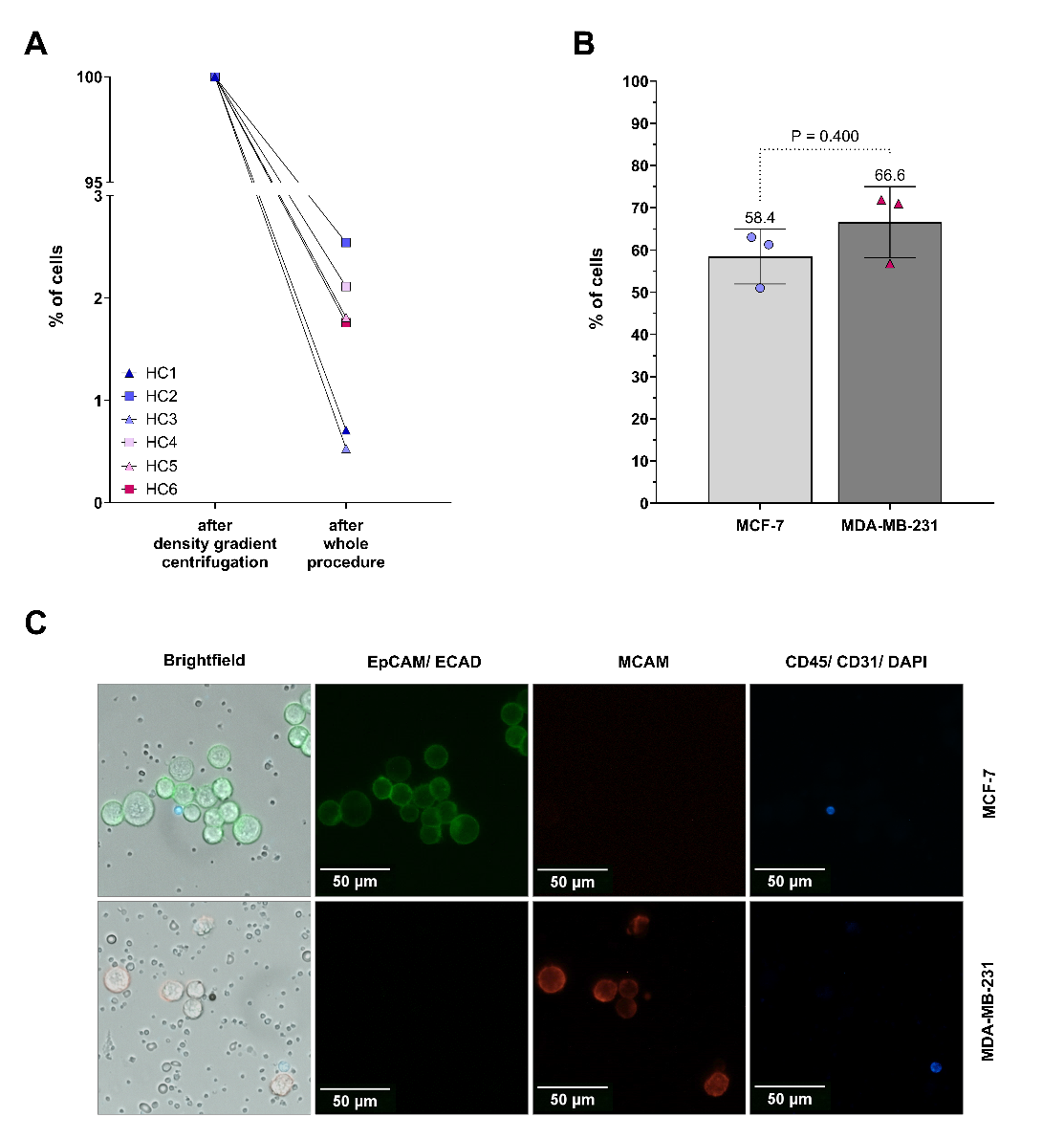
**

**Figure S2 Technical performance of CTCs’ enrichment method.** (**A**) Comparison of the number of PBMCs after density gradient centrifugation and the complete CTCs isolation procedure (analysis of six healthy control (HC) blood samples of 5 ml). (**B**) Recovery rates of 100 spiked MCF-7 (epithelial) and 100 MDA-MB-231 (mesenchymal) cells in 5 mL of blood, presented as mean ± SD from three independent experiments. The groups were compared using Mann-Whitney U test. (**C**) Representative photos of MCF-7 and MDA-MB-231 cells stained for EpCAM, ECAD, MCAM, CD45 and CD31. Cells nuclei were visualized by DAPI staining (based on a figure originally published in Topa J, Żaczek AJ, Markiewicz A. Isolation of Viable Epithelial and Mesenchymal Circulating Tumor Cells from Breast Cancer Patients. Methods Mol Biol. 2024;2752:43-52. doi: 10.1007/978-1-0716-3621-3_3).


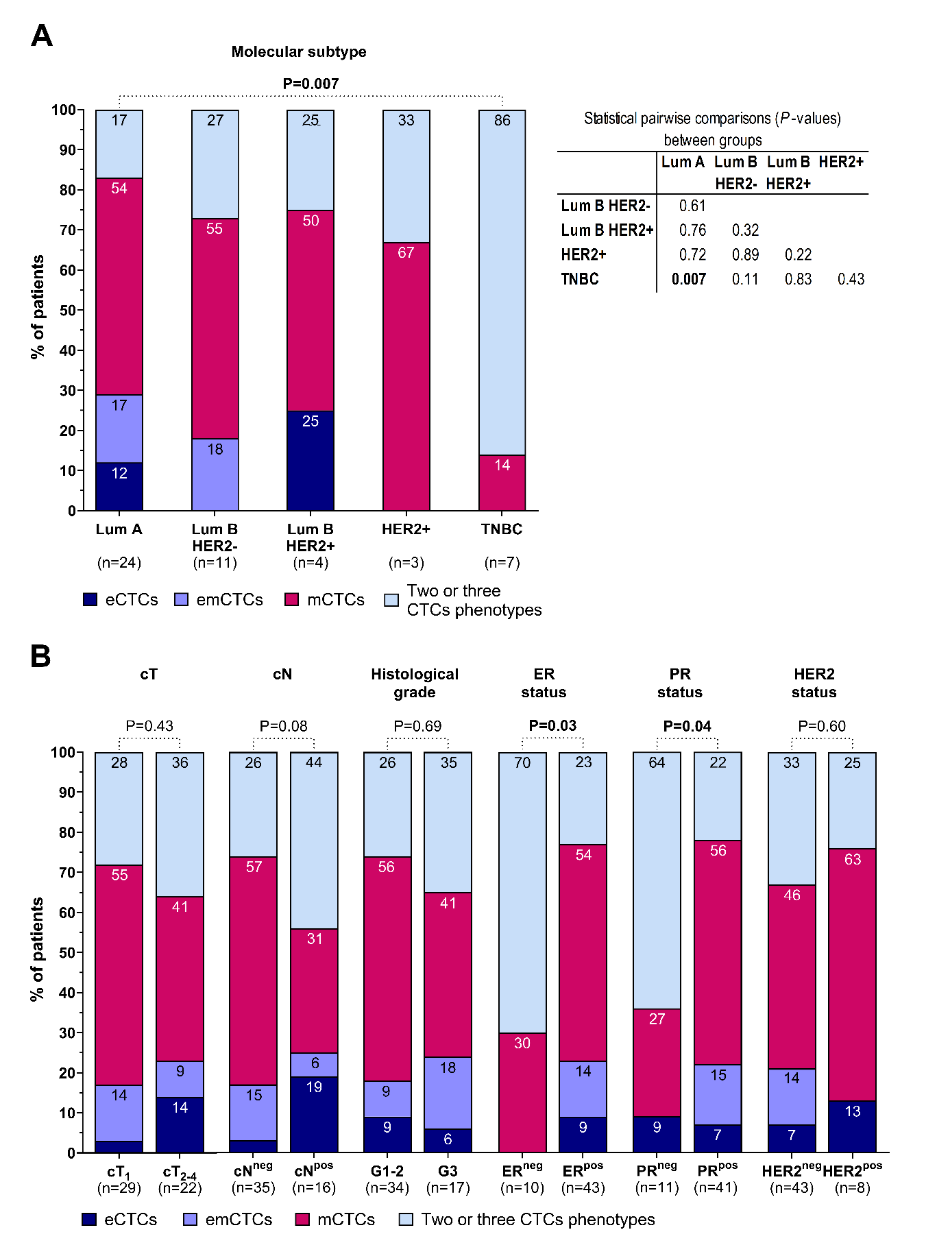


**Figure S3 (A) CTCs phenotypic heterogeneity according to molecular subtype and (B) clinico-pathological characteristics of BC patients.** The variables were compared were using Pearson’s χ2 test. eCTCs – epithelial CTCs, emCTCs – epithelial-mesenchymal CTCs, mCTCs – mesenchymal CTCs.


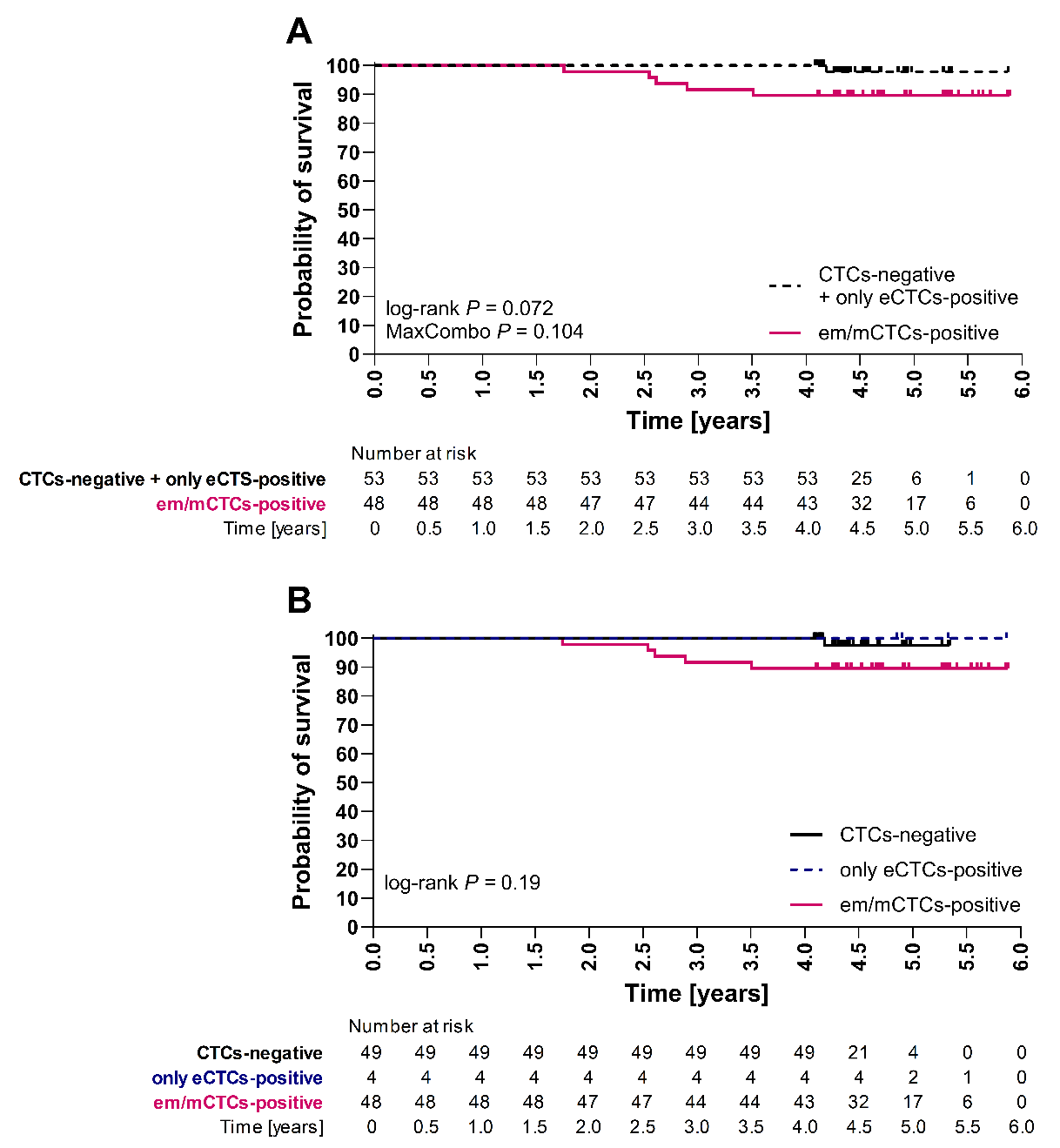


**Figure S4 Survival probability of patients depending on presence and phenotype of detected CTCs.** Kaplan-Meier curves were comparing survival in two groups (A) patients with no CTCs or only epithelial CTCs (eCTCs) vs patients in whom epithelial-mesenchymal (emCTCs) or mesenchymal CTCs (mCTCs) were detected; (B) three groups of patients: patients with no CTCs vs patients with only eCTC vs patients in whom emCTC or mCTCs were detected.


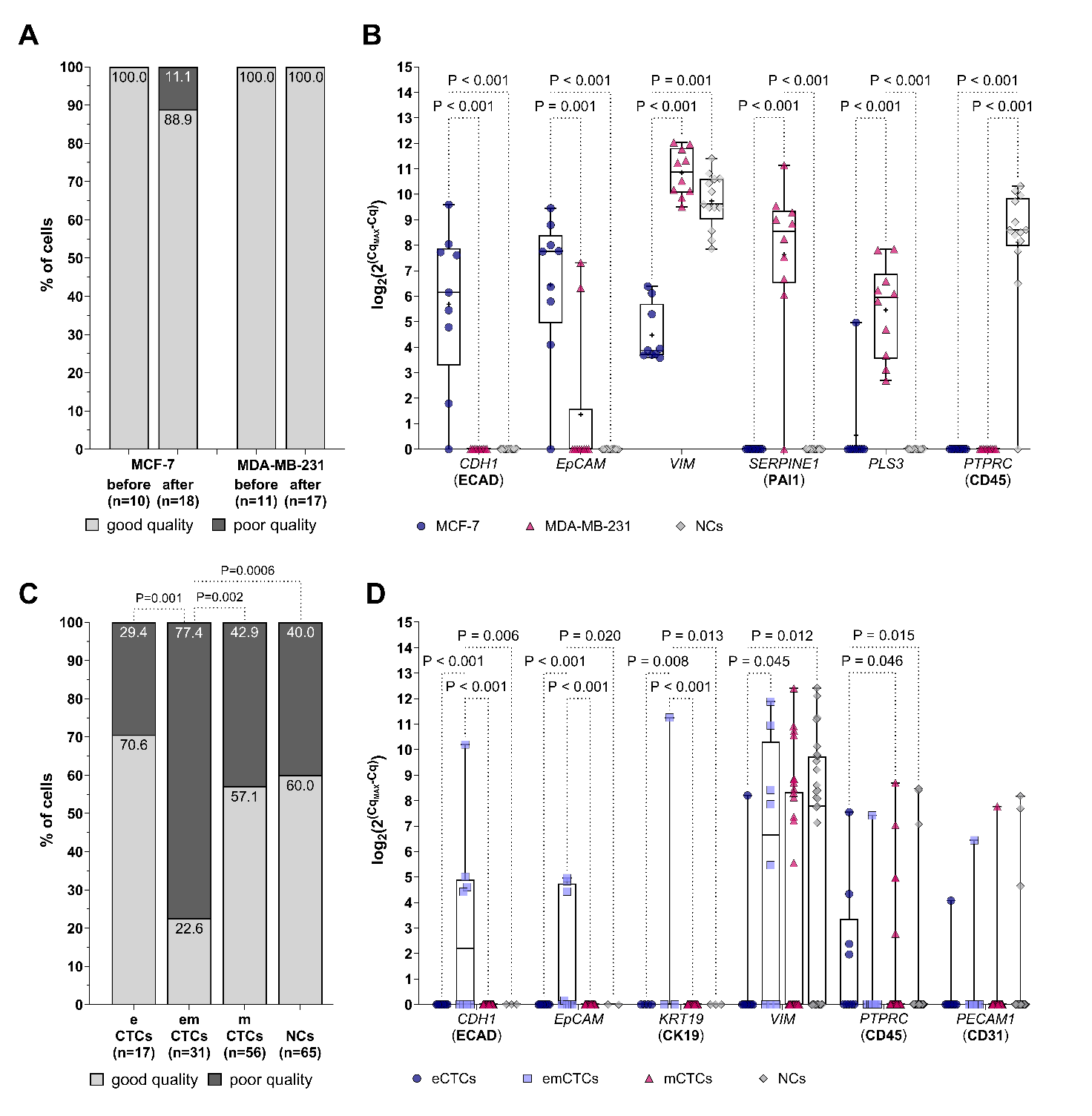


**Figure S5 Transcriptomic characterization of single cells. (A)** The transcriptome quality of post-culture and spiked post-procedure MCF-7 and MDA-MB-231 single cells. (**B**) The expression of epithelial (*CDH1, EpCAM*), mesenchymal (*VIM, PLS3, SERPINE1*), and hematopoietic cell (*CD45*) markers in spiked single MCF-7 and MDA-MB-231 cells and in PBMCs. (**C**) The percentage of the good-quality transcriptome in CTCs by phenotype (n=105) and in normal cells (n=66). (**D**) The expression of epithelial (*CDH1, EpCAM*), mesenchymal (*VIM*), hematopoietic (*CD45*) and endothelial cell (*CD31*) markers in single CTCs and normal cells (PBMCs) from BC patients. The box show interquartile range (25^th^ to 75^th^ percentile) with internal line indicates the median. Only significant differences (*P*<0.05) are plotted. Categorical variables were compared were using Pearson’s χ2 test. Quantitative variables were examined using the Kruskal-Wallis test followed by Dunn’s multiple comparisons test. NCs – normal cells (from the PBMC cell fraction), eCTCs – epithelial CTCs, emCTCs – epithelial-mesenchymal CTCs, mCTCs – mesenchymal CTCs.

**
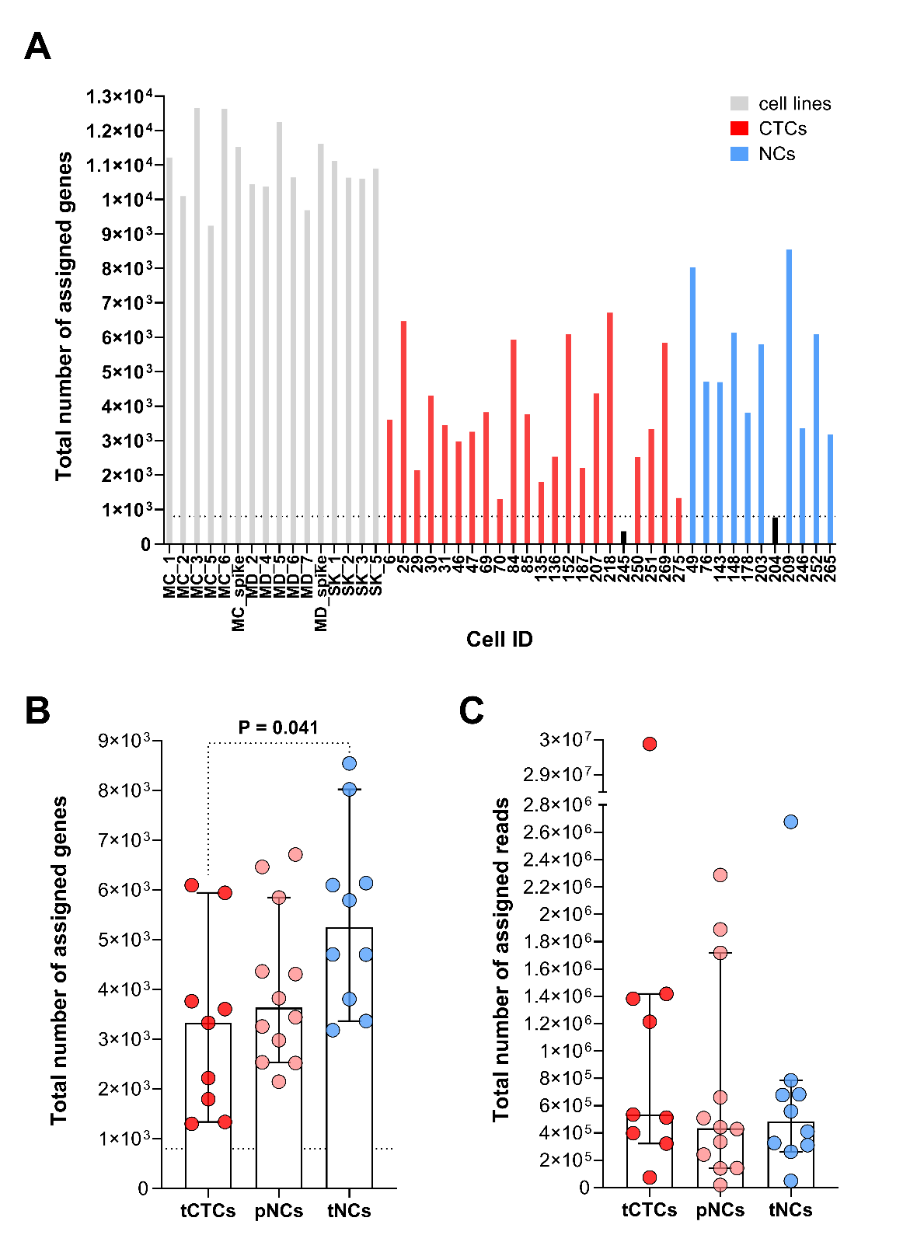
**

**Figure S6 Quality of patients’ CTCs and NCs transcriptome.** (**A**) The number of mapped genes in single cells from BC cell lines (grey) and in single cells captured from BC patients – CTCs (red) and NCs (blue). The cells excluded from further analyses are shown in black. MC – single cells from MCF-7 cell line, MC_spike – single cell from MCF-7 cell line isolated from whole blood (after spike-in test), MD – single cells from MDA MB-231 cell line, MD_spike – single cell from MDA-MB-231 cell line isolated from whole blood (after spike-in test), SK – single cells from SK-BR-3 cell line. (**B**) The number of expressed genes and (**C**) assigned gene reads in single tCTCs (true CTCs – red), pNCs (potential normal cells from the PBMC fraction – light red), tNCs (true normal cells – blue) compared by Kruskal-Wallis test followed by Dunn’s multiple comparisons test. The bar shows median while whiskers show 95% CI. Only significant (*P<*0.05) differences are shown.


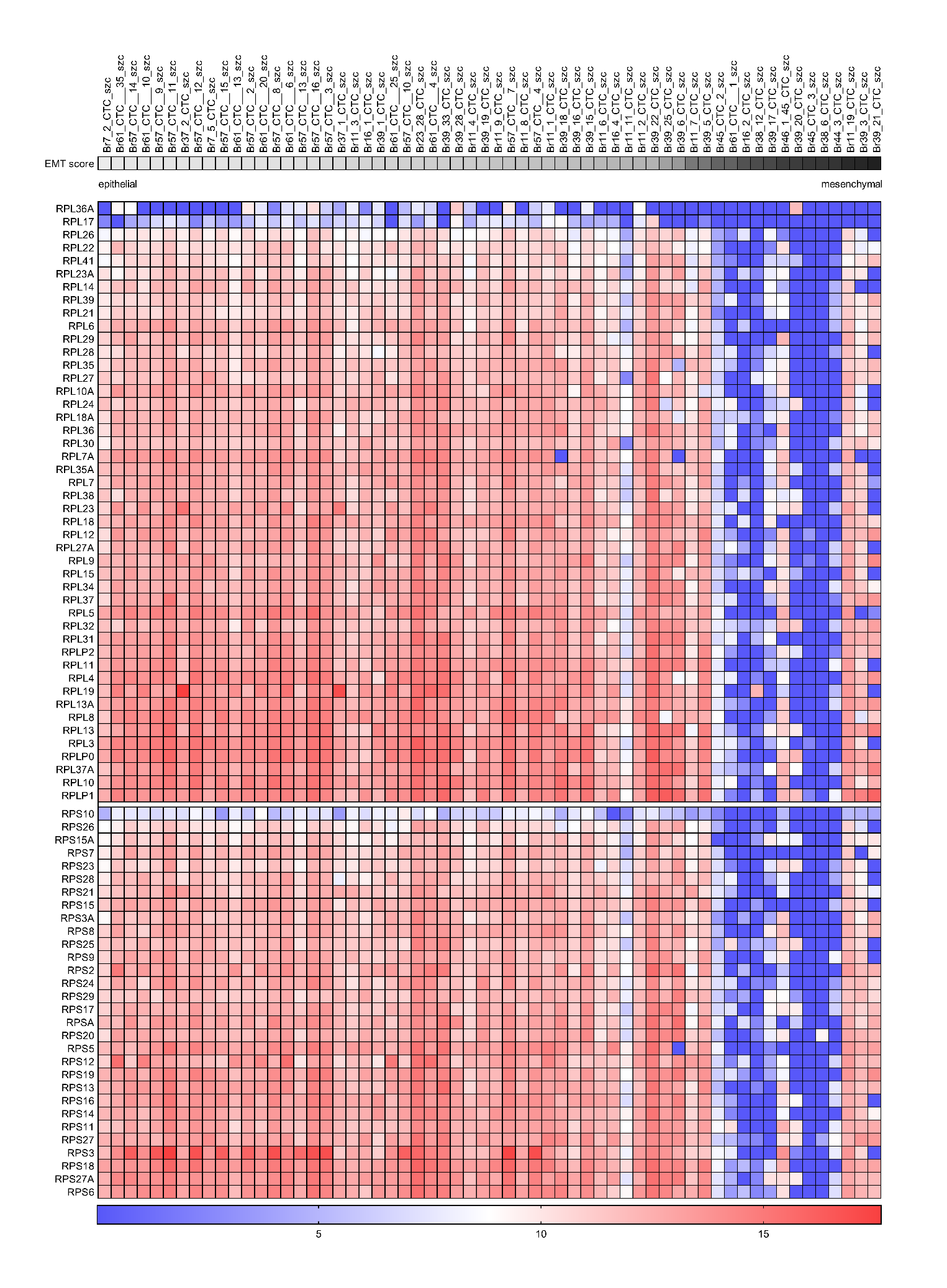


**Figure S7 Gene expression profile of core ribosomal subunits (rows) in single CTCs and WBCs from validation dataset.** Columns represent cell’s ID. EMT score was calculated according to Yu et al. (Science, 2013. 339(6119)).


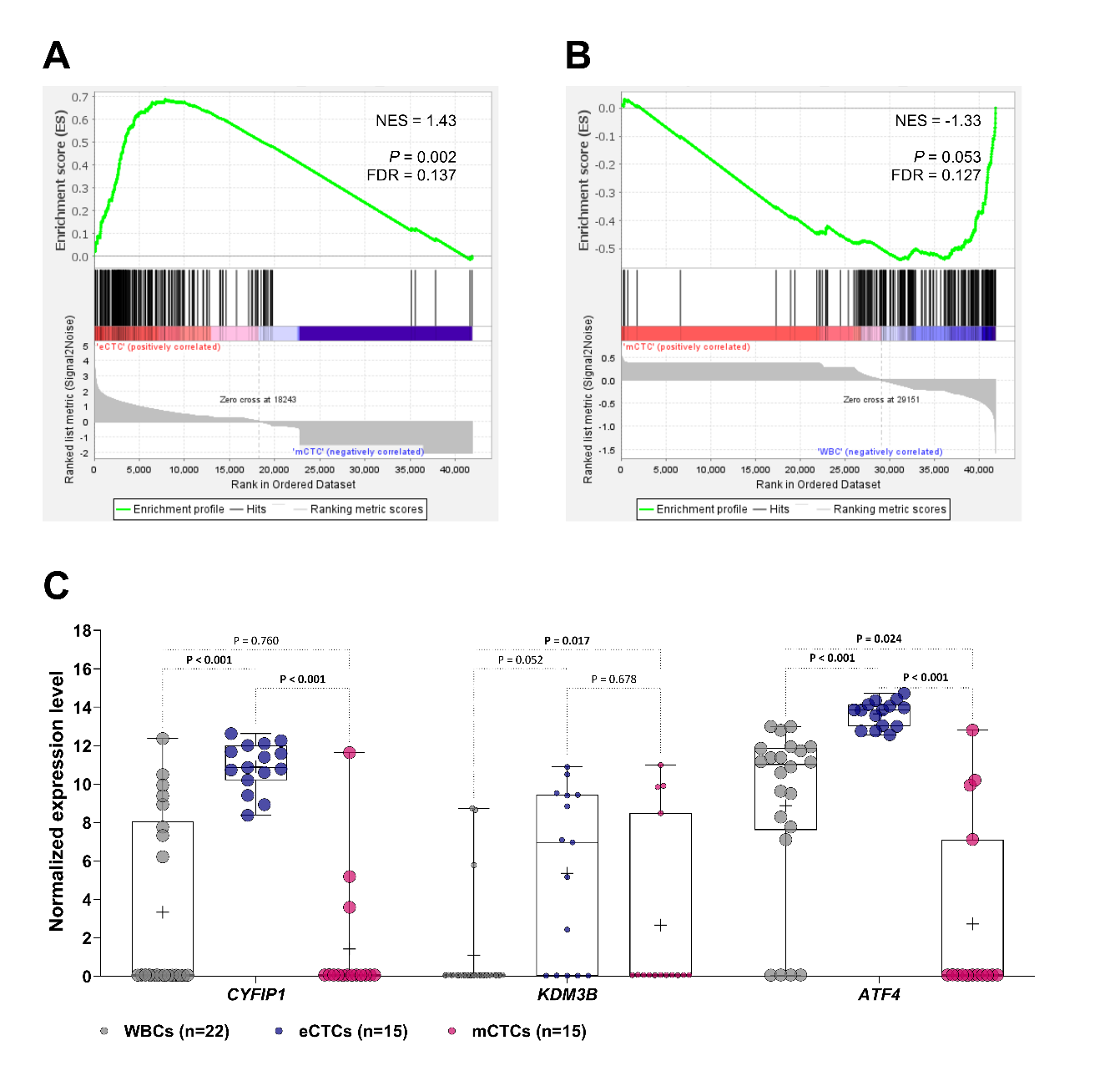


**Figure S8 Gene expression in the validation set.** GSEA analysis of the mTORC1 pathway in **(A)** eCTCs vs WBCs and **(B)** mCTCs vs WBCs. (**C**) Expression of genes identified to be upregulated in our CTCs cohort – *CYFIP1* and *KDM3B*, as well as regulator of stress response - *ATF4*. The box extends from the 25^th^ to 75^th^ percentiles with internal line indicating median and “+” sign indicating mean. The groups were compared using Kruskal-Wallis test followed by Dunn’s multiple comparisons test. WBCs – white blood cells, eCTCs – epithelial CTCs, mCTCs – mesenchymal CTCs.

**Table S1** **Clinico-pathological characteristics of early** **treatment-naïve BC patients (n=104).**

| Characteristic | Status | n | % |  |
| --- | --- | --- | --- | --- |
| Age | <50 years | 24 | 23.1 |  |
|  | ≥50 years | 79 | 75.9 |  |
|  | no data | 1 | 1.0 |  |
| cT  status | cT_1_ | 68 | 65.4 |  |
|  | cT_2_ | 26 | 25.0 |  |
|  | cT_3_ | 3 | 2.9 |  |
|  | cT_4_ | 4 | 3.8 |  |
|  | no data | 3 | 2.9 |  |
| cN  status | cN^neg^ | 81 | 77.9 |  |
|  | cN^pos^ | 20 | 19.2 |  |
|  | no data | 3 | 2.9 |  |
| M  status | M_0_ | 104 | 100 |  |
|  | M_1_ | 0 | 0.0 |  |
|  | no data | 0 | 0.0 |  |
| Histological  grade | G1 | 26 | 25.0 |  |
|  | G2 | 51 | 49.0 |  |
|  | G3 | 24 | 23.1 |  |
|  | no data | 3 | 2.9 |  |
| ER  status | ER^neg^ | 12 | 11.5 |  |
|  | ER^pos^ | 91 | 87.5 |  |
|  | no data | 1 | 1.0 |  |
| PR  status | PR^neg^ | 20 | 19.2 |  |
|  | PR^pos^ | 82 | 78.9 |  |
|  | no data | 2 | 1.9 |  |
| HER2  status | HER2^neg^ | 89 | 85.6 |  |
|  | HER2^pos^ | 10 | 9.6 |  |
|  | no data | 5 | 4.8 |  |
|  | luminal A | 39 | 37.5 |  |
|  | luminal B HER2-negative | 39 | 37.5 |  |
| Molecular subtype | luminal B HER2-positive | 7 | 6.7 |  |
|  | HER2-enriched | 3 | 2.9 |  |
|  | triple-negative | 9 | 8.7 |  |
|  | no data | 7 | 6.7 |  |
| Histological  subtype | ductal | 89 | 85.6 |  |
|  | lobular | 12 | 11.5 |  |
|  | mixed | 1 | 1.0 |  |
|  | no data | 2 | 1.9 |  |

**Table S2 The number and percentage of patients with CTCs with given EMT phenotypes.**

| **CTCs phenotype** | **Number of patients with given**  **CTCs phenotype** | **Percentage of patients with given**  **CTCs phenotype in relation to** | |
| --- | --- | --- | --- |
|  |  | CTCs-positive patients (n=54) | All patients*  (n=104) |
| **eCTCs** | 4 | 7.4 | 3.8 |
| **emCTCs** | 6 | 11.1 | 5.8 |
| **mCTCs** | 27 | 50.0 | 26.0 |
| **Two or three** | 17 | 31.5 | 16.3 |

*includes CTCs-positive (n=54) and CTCs-negative (n=50) patients. eCTCs – epithelial CTCs, emCTCs -epithelial-mesenchymal CTCs, mCTCs – mesenchymal CTCs

**Table S3 The number of CTCs with a given EMT phenotypes in correlation to patients’ clinic-pathological characteristics.**

| **Characteristic** | **Status** | **n** | **any CTCs number** | | | **eCTCs number** | | | **emCTCs number** | | | **mCTCs number** | | |
| --- | --- | --- | --- | --- | --- | --- | --- | --- | --- | --- | --- | --- | --- | --- |
|  |  |  | median | IQR | mean ± SEM | median | IQR | mean ± SEM | median | IQR | mean ± SEM | median | IQR | mean ± SEM |
| **cT**  **status** | cT_1_ | 68 | 0.0 | 0.0-2.1 | 1.8 ± 0.4 | 0.0 | 0.0-0.0 | 0.2 ± 0.1 | 0.0 | 0.0-0.0 | 0.7 ± 0.2 | 0.0 | 0.0-1.0 | 0.9 ± 0.3 |
|  | cT_2-4_ | 33 | 1.3 | 0.0-5.0 | 8.1 ± 4.6 | 0.0 | 0.0-0.0 | 3.0 ± 2.6 | 0.0 | 0.0-.0.9 | 1.5 ± 0.6 | 0.0 | 0.0-1.9 | 3.6± 1.8 |
|  | *P* |  | **0.037** |  |  | 0.094 |  |  | 0.213 |  |  | 0.109 |  |  |
| **cN**  **status** | cN^neg^ | 81 | 0.0 | 0.0-2.1 | 3.5 ± 1.8 | 0.0 | 0.0-0.0 | 1.2 ± 1.1 | 0.0 | 0.0-0.0 | 0.8 ± 0.3 | 0.0 | 0.0-1.0 | 1.5 ± 0.6 |
|  | cN^pos^ | 20 | 1.9 | 0.9-6.3 | 5.2 ± 2.2 | 0.0 | 0.0-1.2 | 1.1 ± 0.5 | 0.0 | 0.0-1.5 | 1.3 ± 0.6 | 0.5 | 0.0-2.9 | 2.8 ± 1.7 |
|  | *P* |  | **0.002** |  |  | **0.002** |  |  | **0.024** |  |  | 0.179 |  |  |
| **Histological grade** | G1-2 | 77 | 0.0 | 0.0-2.0 | 2.2 ± 0.7 | 0.0 | 0.0-0.0 | 0.2 ± 0.1 | 0.0 | 0.0-0.0 | 0.7 ± 0.2 | 0.0 | 0.0-1.0 | 1.3 ± 0.5 |
|  | G3 | 24 | 3.0 | 0.0-6.8 | 9.4 ± 6.0 | 0.0 | 0.0-0.0 | 4.2 ± 3.6 | 0.0 | 0.0-2.1 | 1.7 ± 0.7 | 0.5 | 0.0-3.4 | 3.6 ± 2.0 |
|  | *P* |  | **0.006** |  |  | 0.069 |  |  | 0.057 |  |  |  |  | 0.088 |
| **ER**  **status** | ER^neg^ | 12 | 4.0 | 1.0-7.2 | 15.8 ± 12.0 | 0.5 | 0.0-3.0 | 8.5 ± 7.2 | 0.0 | 0.0-2.1 | 1.8 ± 1.0 | 1.1 | 0.2-3.1 | 5.5 ± 4.0 |
|  | ER^pos^ | 91 | 0.0 | 0.0-2.2 | 2.3 ± 0.6 | 0.0 | 0.0-0.0 | 0.2 ± 0.1 | 0.0 | 0.0-0.0 | 0.8 ± 0.2 | 0.0 | 0.0-1.0 | 1.4 ± 0.4 |
|  | *P* |  | **0.005** |  |  | **<0.001** |  |  | **0.048** |  |  | **0.010** |  |  |
| **PR**  **status** | PR^neg^ | 20 | 1.1 | 0.0-6.8 | 9.8 ± 7.3 | 0.0 | 0.0-1.5 | 5.5 ± 4.3 | 0.0 | 0.0-0.7 | 1.1 ± 0.6 | 0.0 | 0.0-1.5 | 3.3 ± 2.4 |
|  | PR^pos^ | 82 | 0.5 | 0.0-2.3 | 2.3 ± 0.6 | 0.0 | 0.0-0.0 | 0.1 ± 0.0 | 0.0 | 0.0-0.0 | 0.8 ± 0.2 | 0.0 | 0.0-1.0 | 1.5 ± 0.5 |
|  | *P* |  | 0.304 |  |  | **<0.001** |  |  | 0.526 |  |  |  |  | 0.565 |
| **HER2**  **status** | HER2^neg^ | 89 | 0.0 | 0.0-3.0 | 2.1 ± 0.4 | 0.0 | 0.0-0.0 | 0.3 ± 0.1 | 0.0 | 0.0-0.0 | 0.8 ± 0.2 | 0.0 | 0.0-1.1 | 1.1 ± 0.2 |
|  | HER2^pos^ | 10 | 1.5 | 0.7-15.6 | 20.5 ± 14.7 | 0.0 | 0.0-0.5 | 8.9 ± 8.7 | 0.0 | 0.0-2.7 | 2.2 ± 1.5 | 1.0 | 0.0-12.8 | 9.3 ± 5.5 |
|  | *P* |  | 0.119 |  |  | 0.380 |  |  | 0.712 |  |  | **0.024** |  |  |
| **Histological subtype** | ductal | 89 | 1.0 | 0.0-3.6 | 4.4 ± 1.7 | 0.0 | 0.0-0.0 | 1.3 ± 1.0 | 0.0 | 0.0-0.0 | 1.1 ± 0.3 | 0.0 | 0.0-1.4 | 2.0 ± 0.7 |
|  | lobular | 12 | 0.0 | 0.0-1.1 | 1.0 ± 0.7 | 0.0 | 0.0-0.0 | 0.1 ± 0.1 | 0.0 | 0.0-0.0 | 0.2 ± 0.1 | 0.0 | 0.0-0.0 | 0.8 ± 0.7 |
|  | *P* |  | 0.120 |  |  | 0.617 |  |  | 0.466 |  |  | 0.144 |  |  |

The groups were compared using Mann-Whitney U test. An exact *P*-values, which takes into account ties among values, were computed. eCTCs – epithelial CTCs, emCTCs-epithelial-mesenchymal CTCs, mCTCs – mesenchymal CTCs.

**Table S4 Correlation between clinicopathological patients’ characteristics and presence of CTCs with different EMT phenotypes.**

| **Characteristic** | **Status** | **n** | **any CTCs presence** | | **eCTCs presence** | | **emCTCs presence** | | **mCTCs presence** | |
| --- | --- | --- | --- | --- | --- | --- | --- | --- | --- | --- |
|  |  |  | - | + | - | + | - | + | - | + |
| **cT**  **status** | cT_1_ | 68 | 39 | 29 | 62 | 6 | 57 | 11 | 46 | 22 |
|  | cT_2-4_ | 33 | 11 | 22 | 26 | 7 | 24 | 9 | 17 | 16 |
|  | *P* | | **0.02** | | 0.11* | | 0.18 | | 0.12 | |
| **cN**  **status** | cN^neg^ | 81 | 46 | 35 | 75 | 6 | 69 | 12 | 53 | 28 |
|  | cN^pos^ | 20 | 4 | 16 | 13 | 7 | 12 | 8 | 10 | 10 |
|  | *P* | | **0.003** | | **0.003*** | | **0.02*** | | 0.20 | |
| **Histological**  **grade** | G_1-2_ | 77 | 43 | 34 | 71 | 6 | 65 | 12 | 50 | 27 |
|  | G_3_ | 24 | 7 | 17 | 19 | 5 | 16 | 8 | 12 | 12 |
|  | *P* | | **0.02** | | 0.12* | | 0.08* | | 0.19 | |
| **ER**  **status** | ER^neg^ | 12 | 2 | 10 | 6 | 6 | 7 | 5 | 3 | 9 |
|  | ER^pos^ | 91 | 48 | 43 | 84 | 7 | 75 | 16 | 60 | 31 |
|  | *P* | | **0.02*** | | **0.0008*** | | 0.07* | | **0.01*** | |
| **PR**  **status** | PR^neg^ | 20 | 9 | 11 | 13 | 7 | 15 | 5 | 11 | 9 |
|  | PR^pos^ | 82 | 41 | 41 | 76 | 6 | 67 | 15 | 52 | 30 |
|  | *P* | | 0.69 | | **0.003*** | | 0.53* | | 0.49 | |
| **HER2**  **status** | HER2^neg^ | 89 | 46 | 43 | 79 | 10 | 71 | 18 | 58 | 31 |
|  | HER2^pos^ | 10 | 2 | 8 | 8 | 2 | 8 | 2 | 3 | 7 |
|  | *P* | | **0.09*** | | 0.35* | | 1.00* | | **0.04*** | |
| **Histological subtype** | ductal | 89 | 41 | 48 | 78 | 11 | 70 | 19 | 52 | 37 |
|  | lobular | 12 | 8 | 4 | 11 | 1 | 10 | 2 | 10 | 2 |
|  | *P* | | 0.18 | | 1.00* | | 1.00* | | 0.12* | |

The groups were compared using Pearson's χ2 or Fisher's exact test (marked with * sign). eCTCs – epithelial CTCs, emCTCs –epithelial-mesenchymal CTCs, mCTCs – mesenchymal CTCs.

**Table S5 Summary of the regression model selection procedure.**

| **Predictors** | **Model order** | **avg.logLik** | **avg.AIC** | **avg.RMSE** | ***P*** |
| --- | --- | --- | --- | --- | --- |
| cT \| cN \| G \| histological subtype | 5 | -462.47 | 960.95 | 5343.08 | 0.000000 |
| cN \| G \| histological subtype | 4 | -471.94 | 973.87 | 750.96 | 0.000000 |
| cT \| cN \| G | 4 | -479.75 | 989.51 | 2021.54 | 0.000001 |
| cT \| cN \| histological subtype | 4 | -480.13 | 984.25 | 2.40 | 0.000000 |
| cN \| G | 3 | -488.23 | 1000.46 | 681.33 | 0.000000 |
| cN \| histological subtype | 3 | -489.22 | 996.44 | 2.31 | 0.000002 |
| cT \| G \| histological subtype | 4 | -494.89 | 1019.78 | 2.39 | 0.000001 |
| cT \| cN | 3 | -497.7 | 1013.41 | 2.37 | 0.000043 |
| cN | 2 | -504.17 | 1020.34 | 2.30 | 0.000021 |
| cT \| G | 3 | -510.03 | 1044.07 | 2.37 | 0.000023 |
| G \| histological subtype | 3 | -514.62 | 1053.23 | 2.28 | 0.000003 |
| cT \| histological subtype | 3 | -514.89 | 1047.77 | 2.32 | 0.000155 |
| G | 2 | -525.35 | 1068.7 | 2.28 | 0.000074 |
| cT | 2 | -531.76 | 1075.51 | 2.28 | 0.030085 |
| histological subtype | 2 | -538.7 | 1089.39 | 2.24 | 0.000409 |
|  | 1 | -550.1 | 1106.21 | 2.22 | 1.000000 |

The table shows the result of the iterative procedure of searching for the best model, obtained from 500 iterations of calculating avg.logLik, avg.AIC, and avg.RMSE values with a 10-fold cross-validation for all possible models. Levels of statistical significance (*P*) of the models are also provided. Following the exclusion of models comprising the cN/grade (G) combination due to issues with convergence, the top-ranked model includes cT, cN, and histological subtype (cT | cN | histological subtype) as predictors, yielding the highest avg.logLik and lowest avg.AIC, even though the model with cN and histological subtype (cN | histological subtype) alone produced the lowest avg.RMSE. Models are listed in descending order of avg.logLik, possible ties are resolved based on avg.AIC.

**Table S6 The characteristics of the patients from whom single cells were picked.**

| **Cell ID** | **Group of cells** | **Cell phenotype** | **Patient ID** | **cT status** | **cN status** | **M status** | **Treatment** | **ER status** | **PR status** | **HER2 status** | **Molecular subtype** |
| --- | --- | --- | --- | --- | --- | --- | --- | --- | --- | --- | --- |
| 6 | tCTCs | mCTC | BC029 | missing data | missing data | 1 | no | pos | pos | neg | Lum A |
| 70 |  | emCTC | SBC008 | T_2_ | neg | 0 | no | pos | pos | neg | TNBC |
| 84 |  | emCTC | SBC014 | T_1_ | pos | 0 | no | pos | pos | neg | Lum A |
| 85 |  | emCTC | SBC014 | T_1_ | pos | 0 | no | pos | pos | neg | Lum A |
| 135 |  | mCTC | SBC010 | T_1_ | pos | 0 | no | pos | pos | neg | Lum A |
| 152 |  | emCTC | BC084 | T_2_ | pos | 0 | no | pos | neg | neg | LumA |
| 187 |  | mCTC | SBC088 | T_2_ | pos | 0 | no | pos | pos | neg | Lum A |
| 251 |  | mCTC | SBC040 | T_1_ | neg | 0 | no | pos | pos | missing  data | missing  data |
| 275 |  | mCTC | SBC030 | T_1_ | neg | 0 | no | neg | neg | pos | HER2+ |
| 25 | pNCs | emCTC | BC010 | T_1_ | pos | 0 | no | neg | neg | neg | TNBC |
| 29 |  | mCTC | BC011 | T_1_ | pos | 0 | yes | pos | pos | neg | Lum B HER2- |
| 30 |  | mCTC | BC011 | T_1_ | pos | 0 | yes | pos | pos | neg | Lum B HER2- |
| 31 |  | mCTC | BC011 | T_1_ | pos | 0 | yes | pos | pos | neg | Lum B HER2- |
| 46 |  | mCTC | BC033 | missing data | neg | 0 | no | pos | pos | neg | Lum A |
| 47 |  | mCTC | BC033 | missing data | neg | 0 | no | pos | pos | neg | Lum A |
| 69 |  | emCTC | SBC008 | T_2_ | neg | 0 | no | pos | pos | neg | Lum B HER2- |
| 136 |  | mCTC | SBC010 | T_1_ | pos | 0 | no | pos | pos | neg | Lum A |
| 207 |  | mCTC | SBC084 | T_2_ | neg | 0 | no | neg | neg | pos | HER2+ |
| 218 |  | mCTC | SBC063 | T_1_ | pos | 0 | no | pos | pos | neg | Lum A |
| 250 |  | mCTC | SBC040 | T_1_ | neg | 0 | no | pos | pos | missing  data | missing  data |
| 269 |  | emCTC | SBC042 | T_1_ | neg | 0 | no | pos | pos | neg | Lum B HER2- |
| 49 | tNCs | not applicable | BC033 | missing data | neg | 0 | no | pos | pos | neg | Lum A |
| 76 |  |  | SBC009 | T_1_ | neg | 0 | no | pos | pos | neg | Lum A |
| 143 |  |  | BC071 | T_2_ | pos | 0 | yes | pos | pos | neg | Lum B HER2- |
| 148 |  |  | BC079 | T_2_ | pos | 0 | no | pos | pos | pos | Lum B HER2+ |
| 178 |  |  | SBC039 | T_1_ | pos | 0 | no | pos | pos | neg | Lum A |
| 203 |  |  | SBC070 | T_1_ | neg | 0 | no | pos | pos | neg | Lum B HER2- |
| 209 |  |  | SBC084 | T_2_ | neg | 0 | no | neg | neg | pos | HER2+ |
| 246 |  |  | BC098 | T_2_ | neg | 0 | no | pos | pos | pos | Lum B HER2+ |
| 252 |  |  | SBC040 | T_1_ | neg | 0 | no | pos | pos | missing  data | missing  data |
| 265 |  |  | SBC062 | T_1_ | neg | 0 | no | pos | pos | neg | Lum B HER2- |

tCTCs – true CTCs, pNC – potential normal cells, tNCs – true normal cells; eCTCs – epithelial CTCs, emCTCs-epithelial-mesenchymal CTCs, mCTCs – mesenchymal CTCs), neg – negative, pos – positive; Lum A – luminal A; Lum B HER2- luminal B HER2-; Lum B HER2+ – luminal B HER2+; HER2+ – HER2-enriched; TNBC – triple negative breast cancer.

**Table S13 Differences in expression of genes encoding large and small core ribosomal subunits.**

| **Gene name** | **ENSEMBL**  **gene ID** | **Quartiles** | | | | | | | | | ***P*-value** | | |
| --- | --- | --- | --- | --- | --- | --- | --- | --- | --- | --- | --- | --- | --- |
|  |  | **WBCs** | | | **eCTCs** | | | **mCTCs** | | | **WBCs**  **vs**  **eCTCs** | **WBCs**  **vs mCTCs** | **eCTCs**  **vs mCTCs** |
|  |  | Q1 | **Q2** | Q3 | Q1 | **Q2** | Q3 | Q1 | **Q2** | Q3 |  |  |  |
| **Large ribosomal subunits** | | | | | | | | | | | | | |
| **RPL3** | ENSG00000100316.15 | 11.02 | **12.83** | 13.59 | 14.11 | **14.44** | 14.73 | 0.07 | **4.79** | 9.63 | 0.001217 | 0.097297 | 0.000001 |
| **RPL4** | ENSG00000174444.14 | 10.86 | **11.96** | 12.92 | 13.60 | **14.14** | 14.43 | 0.07 | **7.84** | 10.59 | 0.001250 | 0.178917 | 0.000002 |
| **RPL5** | ENSG00000122406.12 | 10.51 | **12.81** | 13.53 | 13.63 | **13.84** | 14.50 | 0.06 | **0.07** | 9.81 | 0.016258 | 0.048073 | 0.000006 |
| **RPL6** | ENSG00000089009.15 | 7.56 | **10.53** | 11.29 | 12.10 | **12.53** | 12.92 | 0.07 | **5.19** | 8.41 | 0.001250 | 0.178917 | 0.000002 |
| **RPL7** | ENSG00000147604.13 | 10.08 | **11.62** | 12.58 | 12.48 | **12.74** | 13.02 | 0.07 | **3.60** | 10.50 | 0.061415 | 0.036849 | 0.000029 |
| **RPL7A** | ENSG00000148303.16 | 10.10 | **12.20** | 12.78 | 13.15 | **13.33** | 13.61 | 0.07 | **0.07** | 9.94 | 0.052403 | 0.078962 | 0.000074 |
| **RPL8** | ENSG00000161016.17 | 10.78 | **12.74** | 13.12 | 13.37 | **14.27** | 14.56 | 0.07 | **3.59** | 11.18 | 0.004357 | 0.016288 | 0.000000 |
| **RPL9** | ENSG00000163682.15 | 9.92 | **12.49** | 12.93 | 12.14 | **12.26** | 12.76 | 1.26 | **6.02** | 10.73 | 1.000000 | 0.065320 | 0.020890 |
| **RPL10** | ENSG00000147403.16 | 10.94 | **13.08** | 13.90 | 13.51 | **14.04** | 14.64 | 0.07 | **7.98** | 11.41 | 0.034670 | 0.049025 | 0.000019 |
| **RPL10A** | ENSG00000198755.10 | 10.26 | **11.36** | 12.05 | 12.29 | **13.12** | 13.41 | 0.07 | **0.07** | 7.66 | 0.003322 | 0.054650 | 0.000001 |
| **RPL11** | ENSG00000142676.12 | 10.66 | **12.85** | 13.69 | 12.94 | **13.37** | 14.02 | 0.07 | **5.78** | 9.24 | 0.482621 | 0.008485 | 0.000172 |
| **RPL12** | ENSG00000197958.12 | 9.88 | **11.68** | 12.66 | 12.18 | **12.77** | 13.46 | 0.07 | **4.29** | 11.62 | 0.032941 | 0.282481 | 0.000330 |
| **RPL13** | ENSG00000167526.13 | 9.83 | **12.48** | 13.31 | 12.79 | **13.85** | 14.48 | 1.27 | **8.28** | 11.96 | 0.024090 | 0.476764 | 0.000591 |
| **RPL13A** | ENSG00000142541.16 | 10.76 | **12.75** | 13.77 | 13.19 | **13.60** | 13.98 | 0.07 | **6.99** | 12.15 | 0.224572 | 0.068148 | 0.000591 |
| **RPL14** | ENSG00000188846.13 | 7.59 | **10.41** | 11.04 | 10.96 | **11.94** | 12.39 | 0.07 | **0.07** | 6.11 | 0.017021 | 0.139856 | 0.000039 |
| **RPL15** | ENSG00000174748.18 | 10.95 | **12.05** | 12.68 | 12.34 | **12.95** | 13.33 | 0.07 | **4.80** | 10.19 | 0.092749 | 0.026076 | 0.000035 |
| **RPL17** | ENSG00000265681.7 | 0.05 | **0.06** | 3.30 | 3.91 | **5.49** | 6.63 | 0.06 | **0.07** | 0.07 | 0.002588 | 1.000000 | 0.002087 |
| **RPL18** | ENSG00000063177.12 | 9.34 | **12.08** | 12.73 | 12.44 | **12.92** | 13.22 | 0.07 | **7.51** | 11.11 | 0.052403 | 0.078962 | 0.000074 |
| **RPL18A** | ENSG00000105640.12 | 8.55 | **10.96** | 11.95 | 11.95 | **12.23** | 12.79 | 2.46 | **6.22** | 9.91 | 0.017947 | 0.156637 | 0.000051 |
| **RPL19** | ENSG00000108298.9 | 10.46 | **12.92** | 14.00 | 12.87 | **13.46** | 14.43 | 0.07 | **5.19** | 11.53 | 0.281075 | 0.012861 | 0.000097 |
| **RPL21** | ENSG00000122026.10 | 9.33 | **10.89** | 11.65 | 10.48 | **10.80** | 11.23 | 0.07 | **5.78** | 9.47 | 1.000000 | 0.012409 | 0.004411 |
| **RPL22** | ENSG00000116251.9 | 6.27 | **8.29** | 9.54 | 10.34 | **10.68** | 11.58 | 0.07 | **2.45** | 8.27 | 0.000179 | 0.600670 | 0.000004 |
| **RPL23** | ENSG00000125691.12 | 9.32 | **12.25** | 12.69 | 12.49 | **12.90** | 13.56 | 0.07 | **7.60** | 10.28 | 0.033167 | 0.122834 | 0.000078 |
| **RPL23A** | ENSG00000198242.13 | 8.39 | **10.51** | 11.08 | 10.25 | **11.27** | 11.95 | 0.07 | **5.78** | 8.51 | 0.175070 | 0.018918 | 0.000067 |
| **RPL24** | ENSG00000114391.12 | 9.65 | **11.10** | 11.82 | 11.71 | **12.04** | 12.34 | 0.07 | **6.73** | 9.16 | 0.021691 | 0.108235 | 0.000035 |
| **RPL26** | ENSG00000161970.12 | 6.05 | **9.77** | 10.62 | 9.35 | **9.75** | 10.66 | 0.07 | **2.46** | 7.96 | 0.948973 | 0.013785 | 0.001305 |
| **RPL27** | ENSG00000131469.12 | 9.08 | **11.57** | 12.58 | 11.02 | **11.66** | 12.09 | 0.07 | **7.84** | 10.38 | 1.000000 | 0.018918 | 0.008432 |
| **RPL27A** | ENSG00000166441.12 | 9.86 | **11.67** | 12.73 | 12.30 | **12.69** | 13.31 | 0.07 | **6.41** | 10.82 | 0.128976 | 0.069222 | 0.000245 |
| **RPL28** | ENSG00000108107.14 | 10.11 | **11.54** | 12.05 | 10.50 | **11.78** | 11.87 | 0.07 | **6.57** | 8.88 | 1.000000 | 0.005050 | 0.002041 |
| **RPL29** | ENSG00000162244.10 | 6.42 | **11.82** | 12.60 | 11.95 | **12.43** | 12.80 | 0.07 | **0.07** | 10.67 | 0.271390 | 0.062002 | 0.000715 |
| **RPL30** | ENSG00000156482.10 | 9.07 | **10.91** | 12.01 | 11.47 | **11.61** | 12.07 | 1.27 | **6.23** | 10.28 | 0.424510 | 0.022003 | 0.000422 |
| **RPL31** | ENSG00000071082.10 | 10.14 | **12.16** | 12.79 | 12.98 | **13.17** | 13.54 | 0.07 | **6.99** | 11.39 | 0.011074 | 0.180866 | 0.000035 |
| **RPL32** | ENSG00000144713.12 | 9.17 | **11.41** | 12.68 | 11.69 | **12.56** | 12.93 | 5.51 | **7.84** | 11.68 | 0.215653 | 0.434296 | 0.008432 |
| **RPL34** | ENSG00000109475.16 | 10.57 | **12.62** | 13.22 | 12.23 | **12.51** | 13.11 | 0.07 | **7.43** | 10.47 | 1.000000 | 0.003721 | 0.001710 |
| **RPL35** | ENSG00000136942.14 | 8.95 | **10.44** | 11.49 | 11.78 | **12.03** | 12.30 | 0.07 | **7.60** | 10.90 | 0.011569 | 0.606987 | 0.000402 |
| **RPL35A** | ENSG00000182899.14 | 2.68 | **11.69** | 12.40 | 12.37 | **12.82** | 13.16 | 0.07 | **6.72** | 11.29 | 0.009030 | 0.308386 | 0.000074 |
| **RPL36** | ENSG00000130255.12 | 8.57 | **10.32** | 11.46 | 12.14 | **12.35** | 12.78 | 0.07 | **5.78** | 11.01 | 0.000806 | 0.701263 | 0.000028 |
| **RPL36A** | ENSG00000241343.9 | 0.05 | **0.05** | 0.06 | 0.03 | **0.03** | 7.35 | 0.06 | **0.06** | 0.07 | 1.000000 | 0.325563 | 0.130797 |
| **RPL37** | ENSG00000145592.13 | 9.86 | **11.92** | 12.54 | 12.32 | **12.72** | 13.02 | 0.07 | **7.33** | 11.47 | 0.059303 | 0.200012 | 0.000402 |
| **RPL37A** | ENSG00000197756.9 | 9.68 | **12.47** | 13.05 | 12.98 | **13.58** | 13.87 | 4.39 | **8.79** | 12.41 | 0.013914 | 0.428892 | 0.000245 |
| **RPL38** | ENSG00000172809.12 | 10.49 | **11.36** | 12.15 | 11.93 | **12.32** | 12.78 | 0.07 | **7.00** | 9.46 | 0.133088 | 0.000866 | 0.000001 |
| **RPL39** | ENSG00000198918.7 | 7.62 | **10.30** | 11.23 | 10.63 | **11.20** | 11.66 | 1.27 | **8.04** | 10.30 | 0.124435 | 0.556003 | 0.006125 |
| **RPL41** | ENSG00000229117.8 | 7.42 | **8.61** | 10.04 | 10.22 | **10.73** | 11.25 | 1.26 | **5.51** | 8.14 | 0.002745 | 0.366706 | 0.000025 |
| **RPLP0** | ENSG00000089157.15 | 10.25 | **11.36** | 13.12 | 13.85 | **14.21** | 15.07 | 0.07 | **8.10** | 12.39 | 0.000062 | 1.000000 | 0.000007 |
| **RPLP1** | ENSG00000137818.11 | 11.24 | **12.95** | 13.50 | 13.62 | **14.08** | 14.24 | 2.46 | **8.23** | 13.16 | 0.043915 | 0.806249 | 0.003429 |
| **RPLP2** | ENSG00000177600.8 | 11.11 | **12.57** | 13.16 | 12.54 | **13.09** | 13.56 | 1.26 | **7.33** | 10.65 | 0.168967 | 0.052744 | 0.000258 |
| **Small ribosomal subunits** | | | | | | | | | | | | | |
| **RPS2** | ENSG00000140988.15 | 9.98 | **11.59** | 12.46 | 12.12 | **12.87** | 13.55 | 0.07 | **5.19** | 10.12 | 0.042909 | 0.188841 | 0.000233 |
| **RPS3** | ENSG00000149273.14 | 11.41 | **13.09** | 13.35 | 13.73 | **14.61** | 16.38 | 0.07 | **7.68** | 10.81 | 0.007707 | 0.059114 | 0.000003 |
| **RPS3A** | ENSG00000145425.9 | 8.91 | **11.56** | 12.05 | 11.57 | **12.00** | 12.47 | 0.07 | **4.28** | 10.51 | 0.399395 | 0.031615 | 0.000591 |
| **RPS5** | ENSG00000083845.8 | 10.74 | **12.19** | 13.27 | 12.75 | **13.26** | 13.88 | 0.07 | **0.07** | 12.09 | 0.033054 | 0.189094 | 0.000163 |
| **RPS6** | ENSG00000137154.12 | 11.20 | **12.85** | 13.28 | 13.49 | **13.85** | 14.26 | 3.02 | **7.11** | 12.06 | 0.010833 | 0.087652 | 0.000009 |
| **RPS7** | ENSG00000171863.12 | 7.34 | **10.78** | 11.35 | 11.37 | **12.39** | 12.93 | 0.06 | **0.07** | 8.06 | 0.004663 | 0.046676 | 0.000001 |
| **RPS8** | ENSG00000142937.11 | 9.26 | **10.64** | 11.99 | 11.73 | **12.19** | 12.74 | 0.07 | **5.51** | 11.11 | 0.026490 | 0.312563 | 0.000299 |
| **RPS9** | ENSG00000170889.13 | 11.19 | **12.68** | 12.84 | 12.13 | **12.65** | 13.01 | 0.07 | **6.72** | 10.14 | 1.000000 | 0.005466 | 0.001138 |
| **RPS10** | ENSG00000124614.13 | 3.58 | **4.53** | 5.18 | 6.07 | **7.20** | 7.62 | 0.07 | **0.07** | 4.54 | 0.002745 | 0.366706 | 0.000025 |
| **RPS11** | ENSG00000142534.6 | 10.24 | **12.29** | 13.00 | 11.77 | **12.71** | 13.07 | 0.07 | **10.05** | 12.24 | 0.925801 | 0.051311 | 0.005421 |
| **RPS12** | ENSG00000112306.7 | 9.26 | **12.49** | 13.48 | 12.00 | **13.05** | 14.05 | 1.26 | **6.72** | 11.89 | 0.578938 | 0.097008 | 0.004792 |
| **RPS13** | ENSG00000110700.6 | 10.08 | **12.52** | 13.49 | 13.14 | **13.36** | 13.67 | 0.07 | **5.18** | 11.29 | 0.097734 | 0.043123 | 0.000078 |
| **RPS14** | ENSG00000164587.11 | 10.55 | **12.65** | 13.18 | 12.72 | **12.90** | 13.38 | 0.07 | **2.45** | 10.01 | 0.608892 | 0.004615 | 0.000140 |
| **RPS15** | ENSG00000115268.9 | 8.43 | **11.18** | 11.84 | 12.50 | **12.95** | 13.02 | 0.07 | **0.07** | 8.72 | 0.003138 | 0.167813 | 0.000006 |
| **RPS15A** | ENSG00000134419.15 | 7.44 | **9.84** | 10.85 | 10.38 | **11.44** | 11.76 | 0.07 | **8.05** | 9.75 | 0.008771 | 0.322616 | 0.000078 |
| **RPS16** | ENSG00000105193.8 | 10.25 | **12.09** | 13.07 | 12.78 | **13.41** | 13.77 | 0.07 | **6.72** | 10.49 | 0.078358 | 0.028207 | 0.000029 |
| **RPS17** | ENSG00000182774.10 | 8.86 | **10.84** | 12.02 | 12.11 | **12.38** | 12.57 | 3.24 | **8.34** | 10.36 | 0.007221 | 0.133277 | 0.000011 |
| **RPS18** | ENSG00000231500.6 | 11.10 | **12.72** | 13.23 | 13.53 | **13.95** | 14.47 | 1.27 | **7.42** | 12.45 | 0.000868 | 0.466775 | 0.000011 |
| **RPS19** | ENSG00000105372.6 | 10.37 | **11.63** | 12.86 | 12.68 | **13.12** | 13.75 | 0.07 | **6.22** | 12.09 | 0.006858 | 0.996448 | 0.000682 |
| **RPS20** | ENSG00000008988.9 | 10.45 | **11.85** | 12.81 | 12.03 | **12.46** | 12.71 | 0.07 | **7.91** | 11.50 | 0.555408 | 0.025667 | 0.000863 |
| **RPS21** | ENSG00000171858.17 | 9.30 | **10.80** | 12.00 | 11.67 | **11.90** | 12.71 | 0.07 | **5.18** | 8.83 | 0.055715 | 0.060543 | 0.000054 |
| **RPS23** | ENSG00000186468.12 | 7.79 | **10.75** | 11.28 | 10.29 | **11.17** | 11.53 | 0.07 | **7.11** | 9.81 | 0.362825 | 0.215936 | 0.006377 |
| **RPS24** | ENSG00000138326.18 | 9.60 | **11.92** | 12.66 | 11.90 | **12.74** | 13.16 | 1.26 | **7.11** | 10.17 | 0.140054 | 0.012765 | 0.000026 |
| **RPS25** | ENSG00000118181.10 | 10.79 | **11.87** | 12.68 | 11.45 | **12.00** | 12.49 | 1.27 | **5.19** | 10.55 | 1.000000 | 0.001694 | 0.000285 |
| **RPS26** | ENSG00000197728.9 | 7.41 | **8.95** | 10.10 | 9.89 | **10.46** | 11.05 | 0.07 | **0.07** | 7.17 | 0.131768 | 0.008720 | 0.000014 |
| **RPS27** | ENSG00000177954.11 | 12.08 | **12.71** | 13.66 | 12.60 | **12.90** | 13.16 | 3.38 | **8.98** | 11.99 | 1.000000 | 0.007557 | 0.001788 |
| **RPS27A** | ENSG00000143947.13 | 11.00 | **12.57** | 13.52 | 13.37 | **13.67** | 14.19 | 3.63 | **9.67** | 11.72 | 0.021808 | 0.052829 | 0.000011 |
| **RPS28** | ENSG00000233927.4 | 8.95 | **10.65** | 11.53 | 11.22 | **12.09** | 12.24 | 0.07 | **4.29** | 8.70 | 0.033509 | 0.028012 | 0.000007 |
| **RPS29** | ENSG00000213741.9 | 9.93 | **11.46** | 12.34 | 11.21 | **11.59** | 12.25 | 3.02 | **6.22** | 11.11 | 1.000000 | 0.019793 | 0.003891 |
| **RPSA** | ENSG00000168028.13 | 10.69 | **11.29** | 11.98 | 11.93 | **12.97** | 13.61 | 0.07 | **7.00** | 9.90 | 0.004061 | 0.277238 | 0.000022 |

The groups were compared using Kruskal-Wallis test followed by Dunn’s multiple comparisons test. WBC - white blood cells, eCTCs – epithelial CTCs, mCTCs – mesenchymal CTCs
